# A Network Analysis of Childhood Psychological Abuse and Overgeneralized Autobiographical Memory in Vocational High School Students

**DOI:** 10.3390/bs16101773

**Published:** 2026-09-28

**Authors:** Xinchun Liu, Jiawei Cao, Yijing Zhang, Jingwen Guo, Tingting Tan, Jie Wu

**Affiliations:** 1 School of Education, Tianjin University, Tianjin 300354, China; 2 Mental Health Education Center, Tianjin University, Tianjin 300072, China; 3 Key Research Base of Humanities and Social Sciences of the Ministry of Education, Academy of Psychology and Behavior, Tianjin Normal University, Tianjin 300387, China; 4 Faculty of Psychology, Tianjin Normal University, Tianjin 300387, China; 5 Tianjin Key Laboratory of Student Mental Health and Intelligence Assessment, Tianjin 300387, China

**Keywords:** overgeneralized autobiographical memory, childhood psychological abuse, CaR-FA-X model, rumination, experiential avoidance, network analysis, vocational high school students

## Abstract

**Background:** Overgeneralized autobiographical memory (OGM) has been linked to adolescent psychological difficulties. The CaR-FA-X model proposes that capture and rumination, functional avoidance, and impaired executive control contribute to OGM, and childhood psychological abuse may be embedded in these same processes. **Methods:** A total of 1364 vocational high school students completed measures of childhood psychological abuse, OGM, ruminative responses, experiential avoidance, cognitive avoidance, and executive-function difficulties. A 20-node Gaussian graphical model was estimated using a nonparanormal transformation and EBICglasso. Strength, Expected Influence (EI), Bridge Expected Influence (BEI), node predictability, bootstrap stability, node redundancy, sensitivity analyses, OGM-specific connectivity, and exploratory gender differences were examined. **Results:** The primary network retained 98 of 190 possible edges. Symptom Rumination showed the highest EI (1.33), followed by Intimidation (1.15), Avoidance of Threatening Stimuli (1.12), and Contextual Elaboration (1.10). Experiential Avoidance had the highest BEI (0.69), followed by Symptom Rumination (0.43) and Working Memory Difficulties (0.40); however, Experiential Avoidance constituted a singleton community, making its EI and BEI identical by construction. A complementary OGM-specific analysis showed greater direct connectivity with the OGM community for Working Memory Difficulties (0.065) and Symptom Rumination (0.058) than for Experiential Avoidance (0.009). Mean node predictability was R^2^ = 0.68 (range = 0.22–0.84), and Strength, EI, and BEI each showed a CS coefficient of 0.75. Five potentially redundant pairs formed three components, and the resulting 15-node sensitivity network closely reproduced the primary findings. Gender network structure and global strength did not differ significantly in the primary NPN analysis (*p* = 0.166 and *p* = 0.314, respectively), and no edge survived FDR correction. A modest global-strength difference emerged only in the Spearman sensitivity analysis (*p* = 0.044). **Conclusions:** Rumination, experiential avoidance, and working memory difficulties occupied prominent but distinguishable positions in the broader network. The findings are broadly compatible with CaR-FA-X but do not establish causal pathways, and the OGM-specific analysis cautions against interpreting general bridge centrality as direct connectivity with autobiographical memory.

## 1. Introduction

Adolescence is the peak period of onset for mental disorders, with a substantial share of lifetime cases beginning before age 18 (Patel et al., 2007). Problems that emerge at this stage seldom remain contained: they disrupt learning and peer relationships and, in more severe cases, escalate to substance misuse, aggression, or suicidal behavior.

In China, senior secondary education operates under a dual-track streaming system in which “regular senior high schools” and “vocational senior high schools” run in parallel. Policy has long advocated for “roughly equivalent scale of vocational and regular education” (as reflected in the ratio targets of 1983 and 1994), with approximately half of junior high school graduates entering vocational senior high schools to prepare for employment, while the remainder enter regular senior high schools primarily oriented toward college entrance through the gaokao, thereby also giving rise to prominent societal “anxiety over the senior high school entrance examination” (zhongkao anxiety). In terms of training objectives, vocational senior high schools focus on cultivating a labor reserve force for economic construction that possesses both scientific and cultural knowledge and vocational skills, emphasizing mastery of a specific skill; the program generally lasts three years, with a few specialties lasting two years. Chinese vocational high school students face these risks alongside pressures specific to their educational setting. Most enter this three-year post-junior-high track, which runs parallel to academic senior high school, after underperforming in the high school entrance examination; they are then expected to acquire occupational skills, meet academic requirements, and commit to an occupational identity within a compressed timeframe. Compared with their peers in academic senior high schools, they report higher levels of depression, anxiety, and stress and are more often exposed to family adversity and low socioeconomic status (F. Li et al., 2025; Mundy et al., 2023; Yu, 2023). What remains unclear is how early adverse experience is statistically associated with emotional and cognitive difficulties in this group and which processes may be involved in these associations. Identifying structurally prominent processes may help prioritize candidates for longitudinal and intervention research rather than establish causal mechanisms or treatment targets. Overgeneralized autobiographical memory (OGM) refers to a tendency to retrieve broad categories or summaries of personal experience rather than specific events that occurred at a particular time and place (Sumner et al., 2013; Williams, 2006). The ability to retrieve specific autobiographical memories supports problem solving, emotion regulation, future thinking, and the development of a coherent sense of self. Specific memories are not merely more detailed descriptions of past experiences; they are also an important resource for adaptive cognitive activity. Therefore, lower memory specificity may render future imagination more general, impoverished, and lacking in contextual detail. Reduced autobiographical-memory specificity has been observed across several forms of psychopathology, including depression, post-traumatic stress disorder, and borderline personality disorder (Ros et al., 2017; Stange et al., 2013; Stewart et al., 2018). In depression, it has been regarded as an important cognitive vulnerability (Chen et al., 2012; Gibbs & Rude, 2004; King et al., 2010). Similar retrieval difficulties may also interfere with emotion regulation and interpersonal functioning in borderline personality disorder (Fonagy & Bateman, 2016; Renneberg et al., 2005). Among adolescents, OGM has been associated with major depressive disorder, recurrence of depressive episodes, and nonsuicidal self-injury (Champagne et al., 2016; Zhang, 2022). These findings suggest that OGM is not simply a feature of memory performance but is embedded in broader cognitive and emotional processes relevant to adolescent mental health.

The CaR-FA-X model proposed by Williams (2006) is one of the most influential accounts of OGM. It identifies three processes that can interfere with the retrieval of specific autobiographical memories: capture and rumination (CaR), functional avoidance (FA), and impaired executive control (X). During autobiographical memory search, capture occurs when retrieval becomes dominated by abstract, self-referential information, whereas rumination repeatedly draws attention back to the causes, meanings, and consequences of distress. Functional avoidance refers to curtailing detailed retrieval when specific memories are emotionally painful. Executive-control difficulties may further impair the ability to maintain the retrieval goal, inhibit irrelevant information, and move from general autobiographical knowledge toward a specific event. Although conceptually distinct, these processes are expected to operate in combination rather than independently.

Childhood psychological abuse may be especially relevant to this framework. Psychological abuse encompasses repeated verbal or behavioral experiences that threaten a child’s emotional security, self-worth, or psychological development (Baker et al., 2021). Unlike some forms of physical abuse, psychological abuse may leave no visible injury, yet its effects can extend to emotion regulation, cognition, interpersonal functioning, and later mental health. Childhood psychological abuse has been associated with suicidal ideation (M.-J. Li et al., 2021; Wang et al., 2020), depression and anxiety, and executive-function difficulties (M. Li et al., 2016; Rashid et al., 2024). A growing literature also links childhood abuse to reduced autobiographical-memory specificity (Edler et al., 2021; Hakamata et al., 2021). Early adversity may shape autobiographical memory through altered emotion regulation (Berzenski et al., 2019), avoidance of trauma-related material (Tian et al., 2018), and other cognitive processes involved in organizing and retrieving personal memories. OGM has also been implicated in the association between childhood trauma and suicidal ideation (Jiang et al., 2020a) and may hinder the integration of traumatic experiences in post-traumatic stress disorder (Buckley et al., 2000; Hennings et al., 2021).

Childhood trauma has been associated with later executive-function difficulties (Olff et al., 2014), and inhibitory control is thought to help sustain goal-directed autobiographical memory search (Gong et al., 2018). Rumination has been linked to both childhood trauma and suicidal ideation (Y. Cui et al., 2019), and repetitive negative thinking can increase the likelihood of retrieving overgeneral rather than specific autobiographical memories (L.-X. Cui & Huang, 2007). Avoidance may provide a third point of connection. Adolescents exposed to psychological abuse may try to distance themselves from distressing thoughts, feelings, and memories; when persistent, however, experiential or cognitive avoidance can become maladaptive and has been associated with nonsuicidal self-injury and other psychological difficulties (Lin et al., 2020). Psychological abuse has also been associated with loneliness (Zhou et al., 2026), which may co-occur with poorer cognitive control and greater difficulty regulating memory retrieval (Qi et al., 2024). Together, these findings suggest that psychological abuse, rumination, avoidance, executive-control difficulties, and OGM may be better understood as an interconnected system than as a simple sequence of independent predictors.

As a prevalent form of maltreatment, childhood psychological abuse has begun to receive greater attention in recent years. Specific forms include belittling, humiliating, threatening, intimidating, and manipulative behaviors. Such behaviors can damage an individual’s self-esteem, cause emotional distress, and give rise to a range of mental health problems. When these behaviors persist over time and occur repeatedly, they may contribute to mental health problems in children such as low self-esteem, anxiety, and depression, and may even result in various complex physical and mental health problems during adolescence and adulthood, such as aggressive behavior, self-harm, and substance abuse (Baker et al., 2021; Melamed et al., 2024).

Unlike some forms of physical abuse, psychological abuse may leave no visible injury, yet its effects can extend to emotion regulation, cognition, interpersonal functioning, and later mental health. Childhood psychological abuse has been clearly associated with suicidal ideation (M.-J. Li et al., 2021; Wang et al., 2020), depression and anxiety, executive-function difficulties (M. Li et al., 2016; Rashid et al., 2024), and body image disturbance/body image problems (Bödicker et al., 2022). Regarding the prevalence of childhood psychological/emotional abuse in Chinese populations, existing studies vary considerably due to differences in sample types, measurement instruments, and cutoff thresholds. Current research findings indicate that the unidimensional prevalence of childhood psychological abuse among Chinese populations generally falls within the range of 24.6–48.8%, and it is highly comorbid with neglect; however, large-scale national epidemiological data are currently lacking in China.

Overgeneral autobiographical memory (OGM) and dissociation are both commonly observed in trauma- and avoidance-related psychopathology: OGM is regarded as a functionally avoidant retrieval style, referring to individuals’ tendency to retrieve categorical, generalized memories rather than single, specific events, with its core lying in reduced autobiographical-memory specificity; whereas dissociation is defined by the American Psychiatric Association (APA, 2013) as “a disruption of and/or discontinuity in the normal integration of consciousness, memory, identity, emotion, perception, body representation, motor control, and behavior.” Although the two are clinically and theoretically associated they differ fundamentally in their definitional connotations and underlying mechanisms, and therefore should be distinguished rather than conflated.

CaR-FA-X, however, is not the only theoretical framework through which the association between childhood psychological abuse and autobiographical-memory functioning can be understood. From a developmental psychopathology and latent-vulnerability perspective, childhood maltreatment may alter multiple neurocognitive systems, including threat processing, emotion regulation, reward processing, and executive control, in ways that may be adaptive in an adverse environment but confer vulnerability to later psychopathology (Gerin et al., 2019; McCrory et al., 2017). Emotion-regulation and cognitive-vulnerability accounts likewise suggest that the consequences of psychological abuse may emerge through broader patterns of affective dysregulation, negative self-referential processing, and cognitive vulnerability rather than through an OGM-specific pathway alone (Rashid et al., 2024; Stange et al., 2013). These perspectives overlap with, but are not reducible to, the mechanisms proposed by CaR-FA-X. Accordingly, the present study treats CaR-FA-X as a theoretically informative framework for selecting candidate processes, rather than as the only possible explanation for the association between childhood psychological abuse and OGM.

Most previous studies have examined these associations using correlations, regression models, or mediation analyses. Such approaches are well suited to testing predefined pathways but provide less information about how multiple psychological dimensions are conditionally related within the same system. Network analysis offers a complementary approach by representing psychological variables as nodes and their conditional associations as edges (Cai et al., 2020). It can highlight highly connected variables and identify bridge nodes that link otherwise distinct psychological domains. In a cross-sectional network, however, central or bridge nodes should not be treated as causal mechanisms or established intervention targets. Rather, they indicate structurally prominent variables that merit closer examination in longitudinal or experimental studies.

A network perspective may be particularly informative for the CaR-FA-X model because capture and rumination, avoidance, and executive-control difficulties are theoretically distinct but likely to be interdependent. Childhood psychological abuse is also multidimensional. Experiences such as Intimidation, Neglect, Denigration, Interference, and Indulgence may show different associations with autobiographical memory and related processes. Examining these dimensions in a single network can therefore provide a more differentiated account of the psychological context of OGM than analyses based solely on total scale scores. The same approach also allows the overall organization and connectivity of the network to be compared between male and female students.

Accordingly, the present study examined a network comprising childhood psychological abuse, OGM, ruminative responses, experiential avoidance, cognitive avoidance, and executive functioning in vocational high school students. We had four aims. First, we characterized the conditional association network linking childhood psychological abuse, OGM, and the three broad processes specified by the CaR-FA-X model. Second, we examined node centrality to identify variables occupying prominent positions in the network. Third, we assessed Bridge Expected Influence to identify nodes that most strongly linked different psychological communities. Finally, we used the Network Comparison Test to determine whether overall network structure and global network strength differed between male and female students. Because the analysis was exploratory and evidence in this population remains limited, we did not specify directional hypotheses for individual nodes or gender differences.

## 2. Materials and Methods

### 2.1. Participants

A total of 1599 students aged 14–18 years were recruited through convenience sampling from five secondary vocational schools in Tianjin, Hebei, and Shandong between 2024 and 2025. Following eligibility and data-quality screening, participants were excluded if their questionnaires showed evident response-quality problems, including invariant or straight-line responding, values outside the prespecified response range of the study measures, or incomplete or internally inconsistent demographic information. A total of 1364 participants were retained in the final analytic sample (85.30% of the recruited sample). The final sample comprised 822 males (60.26%) and 542 females (39.74%), with a mean age of 16.13 years (SD = 0.74). The study materials and procedures were approved by the Research Ethics Committee of Tianjin Normal University (Approval No. 2024120204). All participants provided written informed consent, with additional consent obtained from parents or legal guardians where required. Questionnaires were administered anonymously in classroom group sessions.

### 2.2. Measures

#### 2.2.1. Child Psychological Abuse Scale (CPMS) (Pan et al., 2010)

The CPMS contains 23 items assessing five dimensions of childhood psychological abuse: Intimidation, Neglect, Denigration, Interference, and Indulgence. Items are rated on a five-point scale from 0 to 4, with higher scores indicating greater exposure to psychological abuse. Because the five subscale scores were entered as separate network nodes, internal consistency was evaluated at the subscale level. In the present sample, Cronbach’s α ranged from 0.732 to 0.880 across the five dimensions. McDonald’s ω ranged from 0.882 to 0.919 for the four subscales containing at least three items. For the two-item Indulgence subscale, the Spearman–Brown coefficient was 0.734. Complete node-level reliability estimates are reported in Supplementary Table S2.

#### 2.2.2. Overgeneralized Autobiographical Memory Questionnaire (OGMQ) (Jiang et al., 2020b)

The OGMQ contains 19 items assessing trait-like features of overgeneralized autobiographical memory across four dimensions: Spatiotemporal Construction, Controlled Construction, Contextual Elaboration, and Emotional Elaboration. Higher scores indicate greater overgeneralized autobiographical-memory tendencies. The four dimension scores were entered as separate network nodes. In the present sample, node-level internal consistency was acceptable, with Cronbach’s α ranging from 0.776 to 0.806 and McDonald’s ω ranging from 0.833 to 0.866. Complete reliability estimates are provided in Supplementary Table S2.

#### 2.2.3. Ruminative Responses Scale (RRS) (Han & Yang, 2009)

The 22-item Ruminative Responses Scale assesses habitual ruminative responding across three dimensions: Symptom Rumination, Brooding, and Reflective Pondering. Items are rated on a four-point scale, with higher scores indicating a greater tendency to ruminate. The three subscale scores were entered as separate network nodes. Internal consistency was good to excellent in the present sample, with Cronbach’s α values ranging from 0.827 to 0.921 and McDonald’s ω values ranging from 0.878 to 0.946 across the three dimensions. Complete node-level estimates are reported in Supplementary Table S2.

#### 2.2.4. Acceptance and Action Questionnaire (AAQ) (Cao et al., 2013)

Experiential avoidance was assessed using the seven-item Acceptance and Action Questionnaire-II. Items are rated on a seven-point scale, with higher scores indicating greater experiential avoidance. The total score was entered as the Experiential Avoidance network node. Internal consistency was excellent in the present sample (Cronbach’s α = 0.927; McDonald’s ω = 0.948). Gender-specific reliability estimates are reported in Supplementary Table S2.

#### 2.2.5. Cognitive Avoidance Questionnaire (CAQ) (Jiao et al., 2017)

The Cognitive Avoidance Questionnaire contains 25 items assessing five forms of cognitive avoidance: Thought Substitution, Transformation of Images into Thoughts, Distraction, Avoidance of Threatening Stimuli, and Thought Suppression. Items are rated on a five-point scale, with higher scores indicating greater use of cognitive-avoidance strategies. The five dimension scores were entered as separate network nodes. Internal consistency was good to excellent across the five dimensions, with Cronbach’s α ranging from 0.854 to 0.903 and McDonald’s ω ranging from 0.897 to 0.933. Complete node-level estimates are reported in Supplementary Table S2.

#### 2.2.6. Adult Executive Functioning Inventory (ADEXI) (Liu et al., 2022)

The ADEXI contains 14 items assessing two dimensions: Working Memory Difficulties and Inhibition Difficulties. Items are rated on a five-point scale, with higher scores indicating greater executive-function difficulties rather than better executive functioning. The two subscale scores were entered as separate network nodes. In the present sample, internal consistency was high for Working Memory Difficulties (Cronbach’s α = 0.911; McDonald’s ω = 0.933) and acceptable for Inhibition Difficulties (α = 0.818; ω = 0.850). Gender-specific reliability estimates are reported in Supplementary Table S2.

Because network estimation was conducted at the subscale level, reliability was evaluated for the individual network nodes rather than relying solely on total-scale coefficients. Full-sample and gender-specific reliability estimates for all 20 nodes are provided in Supplementary Table S2.

### 2.3. Statistical Analysis

Descriptive analyses were conducted to summarize the distributions of the study variables and their bivariate associations. The network comprised 20 nodes representing four OGM dimensions, three ruminative-response dimensions, one experiential-avoidance node, five cognitive-avoidance dimensions, two executive-function dimensions, and five childhood psychological-abuse dimensions. Spearman rank-order correlations were retained for descriptive purposes because several variables showed noticeable skewness; however, the Spearman correlation matrix was not used as the primary input for the Gaussian graphical model.

Because the network was estimated at the subscale level, internal consistency was evaluated separately for each of the 20 network nodes rather than only for the parent instruments. Cronbach’s α was calculated for all nodes, and McDonald’s ω based on polychoric correlations was additionally estimated for nodes containing at least three items. For the two-item Indulgence subscale, the Spearman–Brown coefficient was reported. Node-level reliability and score distributions were also examined separately by gender. As supplementary psychometric diagnostics, confirmatory factor analyses were conducted for the six source instruments according to their prespecified factor structures using WLSMV estimation for ordinal indicators. Gender measurement comparability was further examined using ordered-categorical multigroup models progressing from configural to threshold, metric, and scalar constraints. Model evaluation considered both absolute fit indices and changes in approximate fit across increasingly constrained models.

Before network estimation, potential node redundancy was examined using the goldbricker procedure in the networktools package. Redundancy was evaluated using a significance level of *p* = 0.05, a proportion-of-different-correlations threshold of 0.25, and a minimum zero-order correlation of 0.50. Because the nodes represented theoretically predefined subdimensions of established measures, potentially redundant nodes were not automatically deleted from the primary analysis. Instead, all 20 theory-defined nodes were retained in the primary network, and a separate redundancy-reduced network was estimated as a sensitivity analysis. For the reduced network, overlapping nodes belonging to the same connected Goldbricker component were standardized and summarized by the first principal component (PC1), thereby preserving the shared variance of the component without selecting individual nodes on the basis of their observed centrality.

The primary network was estimated as a Gaussian graphical model using the graphical least absolute shrinkage and selection operator (GLASSO) with the extended Bayesian information criterion (EBIC), implemented in the bootnet and qgraph packages. To accommodate departures from multivariate normality, a nonparanormal transformation was applied before estimation. The EBIC hyperparameter was set to γ = 0.50. The resulting network therefore represents regularized partial correlations between pairs of nodes after conditioning on all other nodes in the network. Small associations were shrunk toward zero to reduce the retention of potentially spurious edges. In the network visualization, blue edges indicate positive conditional associations, red edges indicate negative conditional associations, and edge thickness represents the magnitude of the regularized partial correlation.

Node importance was evaluated using Strength and one-step Expected Influence (EI). Strength represents the sum of the absolute weights of edges incident on a node, whereas EI retains the signs of edge weights and was therefore particularly informative in a network containing both positive and negative associations. Closeness and Betweenness were not used for substantive interpretation because of concerns regarding their stability and interpretability in psychological networks. One-step Bridge Expected Influence (BEI) was additionally calculated using the networktools package to quantify the extent to which each node was connected to nodes outside its own theoretically defined community. Six communities were specified a priori: OGM, Ruminative Responses, Experiential Avoidance, Cognitive Avoidance, Executive Function, and Childhood Psychological Abuse.

Because BEI reflects general cross-community connectivity rather than connectivity specifically with OGM, an additional descriptive OGM-specific analysis was conducted. Regularized partial edges linking Symptom Rumination (X5), Experiential Avoidance (X8), and Working Memory Difficulties (X14) with the four OGM nodes (X1–X4) were extracted. For each candidate process, signed OGM connectivity was calculated as the sum of the four corresponding edge weights, whereas absolute OGM connectivity was calculated as the sum of their absolute values. These indices were treated as descriptive summaries of conditional connectivity rather than as measures of mediation, temporal transmission, or causal influence.

Node predictability was estimated as the proportion of variance in each node explained by its neighboring nodes. Predictability was calculated as R^2^ using EGAnet::network.predictability on the nonparanormal-transformed data and was visualized as rings around the nodes in the primary network figure. Predictability was interpreted as a measure of how strongly a node was statistically accounted for by the other variables included in the network rather than as evidence of causal determination.

Network accuracy and centrality stability were evaluated using bootstrap procedures implemented in bootnet. A nonparametric bootstrap with 1000 replications was used to examine the accuracy of edge-weight estimates and pairwise differences in edge weights, EI, and BEI. A case-dropping bootstrap with 1000 replications was used to evaluate the stability of Strength, EI, and BEI. Correlation-stability (CS) coefficients quantify the maximum proportion of observations that can be removed while retaining a correlation of at least 0.70 with the original estimates in at least 95% of bootstrap samples. CS coefficients above 0.25 were considered acceptable and values of 0.50 or higher preferable.

Two complementary sensitivity analyses were conducted. First, the complete 20-node network was re-estimated using Spearman correlations with otherwise identical EBICglasso settings, allowing direct assessment of whether the principal findings depended on the nonparanormal estimation strategy. Second, a redundancy-reduced network was estimated after replacing connected within-community Goldbricker components with PC1 composite nodes. Robustness was evaluated by comparing common-node edge weights and the rank ordering of Strength, EI, BEI, and predictability between the primary and reduced networks. PC1 composite nodes were not treated as being equivalent to any individual source node.

Finally, gender differences were examined using the Network Comparison Test (NCT). Separate male and female nonparanormal EBICglasso networks were estimated using identical settings. Based on 1000 permutations, the NCT evaluated network structure invariance and global strength invariance. Edge-specific *p* values were FDR-adjusted across the 190 possible edge comparisons. For exploratory node-level comparisons, FDR correction was applied across the 40 requested node-by-centrality tests (20 nodes × Strength and EI). Statistical significance was set at *p* < 0.05, and all gender comparisons were treated as secondary and exploratory analyses.

## 3. Results

### 3.1. Descriptive Statistics and Bivariate Associations

The final analytic sample comprised 1364 vocational high school students (822 male and 542 female students), with complete data on all 20 network nodes. Descriptive statistics are presented in Table 1. Several variables showed appreciable positive skewness, particularly Spatiotemporal Construction, Emotional Elaboration, Neglect, Denigration, and Indulgence, which supported the use of a nonparanormal transformation for the primary Gaussian graphical model.

Spearman correlations were retained to describe zero-order associations. Thirteen pairwise correlations were at least 0.80 in absolute value. The largest were Symptom Rumination–Brooding (ρ = 0.857), Distraction–Avoidance of Threatening Stimuli (ρ = 0.854), Symptom Rumination–Reflective Pondering (ρ = 0.852), and Avoidance of Threatening Stimuli–Thought Suppression (ρ = 0.847). The full zero-order Spearman correlation matrix is presented in Supplementary Table S1. Because these correlations were used descriptively rather than as the primary GGM input, the conditional-dependence results below are based on the nonparanormal-transformed data.

### 3.2. Psychometric Properties and Gender Measurement Comparability

Because the network variables represented individual subscales, internal consistency was evaluated separately for all 20 nodes. Reliability was generally acceptable to excellent. Across the full sample, Cronbach’s α ranged from 0.732 to 0.927. For the 19 nodes containing at least three items, polychoric McDonald’s ω ranged from 0.833 to 0.948. The two-item Indulgence subscale had a Spearman–Brown coefficient of 0.734. Gender-specific reliability estimates were similarly satisfactory: Cronbach’s α ranged from 0.714 to 0.934 among males and from 0.714 to 0.908 among females. In particular, the two ADEXI nodes showed adequate reliability, with α = 0.911 and ω = 0.933 for Working Memory Difficulties and α = 0.818 and ω = 0.850 for Inhibition Difficulties. Complete full-sample and gender-specific estimates are reported in Supplementary Table S2. Gender-specific descriptive statistics for the 20 network nodes are provided in Supplementary Table S3.

Confirmatory factor analyses were conducted as supplementary psychometric diagnostics for the six source instruments. All standardized item loadings were positive and statistically significant, ranging from 0.510 to 0.927, although global model fit varied across measures. The ADEXI showed comparatively satisfactory fit (CFI = 0.951, TLI = 0.942, RMSEA = 0.076, SRMR = 0.034). The OGMQ (CFI = 0.901, TLI = 0.885, RMSEA = 0.090, SRMR = 0.044), RRS (CFI = 0.894, TLI = 0.881, RMSEA = 0.099, SRMR = 0.041), AAQ (CFI = 0.965, TLI = 0.948, RMSEA = 0.125, SRMR = 0.025), and Cognitive Avoidance Questionnaire (CFI = 0.914, TLI = 0.903, RMSEA = 0.091, SRMR = 0.034) showed mixed fit across indices. Global fit was weaker for the childhood psychological abuse measure (CFI = 0.808, TLI = 0.780, RMSEA = 0.136, SRMR = 0.065). These analyses therefore provided supplementary measurement information rather than uniformly confirming optimal factorial fit for all instruments.

For the OGMQ, RRS, AAQ, Cognitive Avoidance Questionnaire, and ADEXI, ordered-categorical multigroup models converged through the scalar level. From the threshold model through the metric and scalar models, changes in approximate fit were small (|ΔCFI| ≤ 0.003, maximum increase in RMSEA = 0.0014, and |ΔSRMR| < 0.001), providing approximate support for the comparability of factor loadings and intercepts across gender. Several scaled chi-square difference tests were nevertheless statistically significant, and absolute configural-model fit was not uniformly strong; measurement equivalence was therefore interpreted cautiously rather than as definitive full invariance. For the childhood psychological abuse measure, the complete invariance sequence could not be established because the configural and scalar models did not converge successfully, although gender-specific node reliabilities remained satisfactory. Detailed CFA fit indices and standardized factor loadings are presented in Supplementary Tables S4 and S5, respectively, and the gender measurement-invariance results are reported in Supplementary Table S6.

### 3.3. Primary Network Structure and Edge Characteristics

The primary nonparanormal EBICglasso network comprised 20 nodes and 190 possible undirected edges (Figure 1). After regularization, 98 edges remained nonzero, including 80 positive and 18 negative edges, yielding a network density of 0.516 and a global strength of 9.618. Twenty-nine retained edges connected nodes within the same predefined community, whereas 69 connected nodes from different communities.

The strongest positive edge connected Working Memory Difficulties (X14) and Inhibition Difficulties (X15; w = 0.492), followed by Symptom Rumination (X5) and Brooding (X6; w = 0.465), Symptom Rumination and Reflective Pondering (X7; w = 0.436), Intimidation (X16) and Denigration (X18; w = 0.409), Spatiotemporal Construction (X1) and Emotional Elaboration (X4; w = 0.400), and Controlled Construction (X2) and Contextual Elaboration (X3; w = 0.390). The strongest cross-community associations were Experiential Avoidance (X8)–Working Memory Difficulties (X14; w = 0.174), Symptom Rumination (X5)–Experiential Avoidance (X8; w = 0.163), and Inhibition Difficulties (X15)–Intimidation (X16; w = 0.098).

Negative edges were generally small. The largest negative conditional associations were Emotional Elaboration (X4)–Inhibition Difficulties (X15; w = −0.051), Experiential Avoidance (X8)–Denigration (X18; w = −0.043), and Denigration (X18)–Indulgence (X20; w = −0.035). Direct conditional associations between childhood psychological abuse and OGM remained sparse: only four abuse–OGM edges were nonzero, and all had absolute weights of 0.026 or smaller. Thus, the primary analysis continued to show little evidence of strong direct conditional connectivity between the childhood-psychological-abuse and OGM communities.

### 3.4. Node Centrality and Predictability

Strength and Expected Influence (EI) are presented in Figure 2 and Table 2. Symptom Rumination (X5) had the highest Strength (1.328) and EI (1.328). The next highest Strength values were observed for Intimidation (X16; 1.148), Denigration (X18; 1.134), Avoidance of Threatening Stimuli (X12; 1.132), and Contextual Elaboration (X3; 1.130). For EI, Symptom Rumination was followed by Intimidation (1.148), Avoidance of Threatening Stimuli (1.118), Contextual Elaboration (1.098), and Neglect (X17; 1.066).

Node predictability ranged from R^2^ = 0.221 to 0.841, with a mean R^2^ of 0.679. Symptom Rumination had the highest predictability (R^2^ = 0.841), followed by Avoidance of Threatening Stimuli (R^2^ = 0.824) and Distraction (X11; R^2^ = 0.818). Indulgence (X20) had the lowest predictability (R^2^ = 0.221), followed by Inhibition Difficulties (X15; R^2^ = 0.489) and Controlled Construction (X2; R^2^ = 0.519). These estimates represent within-sample statistical predictability from neighboring variables and should not be interpreted as out-of-sample prediction or causal controllability.

### 3.5. Bridge Expected Influence and OGM-Specific Connectivity

Bridge Expected Influence (BEI) was used to characterize signed connectivity with nodes outside each node’s predefined community. Experiential Avoidance (X8) had the highest estimated BEI (0.688), followed by Symptom Rumination (X5; 0.427) and Working Memory Difficulties (X14; 0.404). Intimidation (X16; 0.240), Inhibition Difficulties (X15; 0.188), Thought Suppression (X13; 0.172), Neglect (X17; 0.165), and Avoidance of Threatening Stimuli (X12; 0.164) had the next highest values (Figure 3).

Experiential Avoidance requires a specific interpretive qualification because it was the sole node in its predefined community. Consequently, every edge incident on Experiential Avoidance was, by definition, a cross-community edge, and its EI and BEI were therefore identical by construction (EI = BEI = 0.688). Its high BEI should accordingly be interpreted as broad connectivity with the rest of the network rather than as evidence that Experiential Avoidance is specifically connected with the OGM community.

To distinguish general cross-community prominence from connectivity specifically with OGM, we conducted a complementary descriptive analysis of the regularized partial edges linking Symptom Rumination, Experiential Avoidance, and Working Memory Difficulties with the four OGM nodes. Working Memory Difficulties showed the largest absolute OGM-specific connectivity (0.065), followed by Symptom Rumination (0.058), whereas Experiential Avoidance showed only minimal OGM-specific connectivity (0.009). For Working Memory Difficulties, the retained OGM connection was with Spatiotemporal Construction (w = 0.065). Symptom Rumination showed small positive conditional connections with Spatiotemporal Construction (w = 0.028), Controlled Construction (w = 0.027), and Contextual Elaboration (w = 0.002). Experiential Avoidance showed only small negative connections with Emotional Elaboration (w = −0.007) and Contextual Elaboration (w = −0.002), with no retained edges to the other two OGM nodes.

The same ordering was observed in the Spearman sensitivity network, in which absolute OGM-specific connectivity was 0.079 for Working Memory Difficulties, 0.052 for Symptom Rumination, and 0.005 for Experiential Avoidance. These OGM-specific connectivity indices are descriptive summaries of conditional edge weights and should not be interpreted as mediation, temporal transmission, or causal effects. Detailed OGM-specific edge weights and connectivity indices are presented in Supplementary Table S7 and Figure S6.

### 3.6. Network Accuracy and Stability

The nonparametric bootstrap indicated that larger edge estimates were generally more precisely estimated than weak or near-zero edges (Figure 4). Accordingly, substantive interpretation emphasized larger conditional associations and broader node-level patterns rather than small numerical differences among weak edges. Detailed bootstrap confidence intervals for edge weights are shown in Supplementary Figure S2, and bootstrap difference tests for edge weights, Expected Influence, and Bridge Expected Influence are presented in Supplementary Figure S3A–C.

The case-dropping bootstrap demonstrated preferable stability for all three focal node-level indices. The correlation-stability coefficients were 0.750 for Strength, 0.750 for Expected Influence, and 0.750 for Bridge Expected Influence. Thus, the rankings of centrality and bridge-centrality estimates remained strongly correlated with the full-sample estimates after substantial case removal.

### 3.7. Node Redundancy and Sensitivity Analyses

Goldbricker identified five potentially redundant node pairs, all within their predefined theoretical communities. These pairs formed three connected components: (1) Spatiotemporal Construction (X1) and Contextual Elaboration (X3); (2) Brooding (X6) and Reflective Pondering (X7); and (3) Thought Substitution (X9), Transformation of Images into Thoughts (X10), Avoidance of Threatening Stimuli (X12), and Thought Suppression (X13) (Table 3). The Cognitive-Avoidance component comprised Thought Substitution, Transformation of Images into Thoughts, Avoidance of Threatening Stimuli, and Thought Suppression; Distraction (X11) was not included in this redundancy component. The individual Goldbricker-identified redundancy candidate pairs are reported in Supplementary Table S8.

Because these variables represented theory-defined subdimensions of established instruments, all 20 original nodes were retained in the primary network. A separate redundancy-reduced sensitivity network was estimated by replacing each connected Goldbricker component with its standardized first principal component. The PC1 composites explained 86.7% of the variance in the OGM component, 90.8% in the ruminative-response component, and 84.9% in the cognitive-avoidance component. This procedure yielded a 15-node reduced network containing 56 nonzero edges. The redundancy-reduced sensitivity analyses are illustrated in Supplementary Figure S4A–D.

Among the 12 unchanged original nodes that could be compared directly between the primary and reduced networks, edge weights were nearly identical (Pearson r = 0.999; Spearman r_s_ = 0.979). The Jaccard overlap of nonzero common-node edges was 0.923, and the sign of every common edge that was nonzero in both networks was preserved. Rank correlations across unchanged nodes were r_s_ = 0.951 for Strength, 0.979 for Expected Influence, 0.993 for Bridge Expected Influence, and 1.000 for predictability.

The separate 20-node Spearman EBICglasso sensitivity network also closely reproduced the primary NPN network. Across all 190 possible edges, Pearson and Spearman correlations of edge weights were 0.998 and 0.963, respectively, and the Jaccard overlap of nonzero edges was 0.868. Rank correlations between the NPN and Spearman networks were r_s_ = 0.979 for Strength, 0.995 for Expected Influence, and 0.991 for Bridge Expected Influence. Together, the sensitivity analyses indicated that the principal node-level findings were highly robust to both the correlation–estimation strategy and the treatment of Goldbricker-flagged redundancy (shown in Table 4). The Spearman sensitivity network and the NPN-versus-Spearman edge-weight comparison are shown in Supplementary Figure S1A,B.

### 3.8. Gender Network Comparison

The gender comparison was treated as a secondary and exploratory analysis (Table 5). In the primary NPN Network Comparison Test, the overall network structure did not differ significantly between male and female students (M = 0.151, *p* = 0.166). Importantly, the Global Strength Invariance Test was also nonsignificant (S = 0.238, *p* = 0.314), although the estimated global strength was numerically higher in males (9.313) than in females (9.075). None of the 190 edge-specific comparisons survived FDR correction (minimum q = 0.295). The male and female primary NPN networks are displayed in Supplementary Figure S5A,B.

Exploratory node-centrality comparisons, with FDR correction across the 40 requested node-by-centrality tests, identified four differences in the primary NPN analysis. Interference (X19) showed higher Strength in males than females (0.942 vs. 0.698; q = 0.040). Expected Influence was higher in males for Interference (0.932 vs. 0.641; q = 0.020) and Indulgence (X20; 0.371 vs. 0.212; q = 0.020), whereas Experiential Avoidance showed higher EI in females than males (0.865 vs. 0.630; q = 0.050). These isolated node-level findings should be interpreted cautiously because neither overall network structure nor global strength differed significantly and no individual edge difference survived FDR correction. Complete FDR-adjusted node-centrality comparisons are reported in Supplementary Table S9.

The Spearman-based gender sensitivity analysis likewise showed no significant network-structure difference (M = 0.145, *p* = 0.219), and no edge-specific comparison survived FDR correction (minimum q = 0.391). In contrast to the primary NPN analysis, the Spearman sensitivity analysis yielded a modest global-strength difference (male = 9.392, female = 8.890; S = 0.502, *p* = 0.044). Because this global-strength result was not reproduced by the prespecified primary NPN analysis, evidence for a gender difference in overall network connectivity was considered estimation-dependent and therefore not robust. The results consequently provide stronger support for overall similarity than for systematic gender differences in network organization or connectivity. Full edge-specific Network Comparison Test results with FDR-adjusted *p* values for both the primary NPN and Spearman analyses are provided in Supplementary Data S1.

## 4. Discussion

The present study examined childhood psychological abuse, overgeneralized autobiographical memory (OGM), and CaR-FA-X-related processes within a single conditional-dependence network and subjected the principal findings to several complementary robustness checks. Four findings were especially notable. First, Symptom Rumination showed the highest Expected Influence and also the highest node predictability, with Intimidation occupying another consistently prominent position. Although Avoidance of Threatening Stimuli and Contextual Elaboration also showed high EI in the full 20-node network, both belonged to Goldbricker-identified redundancy components and their individual rankings therefore warrant greater caution. Second, Experiential Avoidance, Symptom Rumination, and Working Memory Difficulties showed the three highest Bridge Expected Influence values, and this bridge ranking was highly stable across case-dropping, correlation-strategy, and redundancy-reduction analyses. Third, the redundancy-reduced network closely reproduced the edge structure and node rankings of the primary network, suggesting that the main conclusions were not artifacts of topological overlap. Finally, male and female students showed broadly similar network configurations, and the primary NPN analysis did not identify a significant difference in global strength; a modest global-strength difference emerged only in the Spearman sensitivity analysis. Because this difference was not reproduced by the primary model, the gender findings provide stronger evidence for overall similarity than for systematic differences in network connectivity.

### 4.1. Core Nodes and the CaR-FA-X Framework

Symptom Rumination emerged as the most consistently prominent individual node. It had the highest Strength and Expected Influence in the primary network, the highest node predictability, and retained the highest EI among unchanged nodes in the Goldbricker-reduced sensitivity analysis. This pattern is consistent with the capture-and-rumination component of the CaR-FA-X model (Williams, 2006). During autobiographical-memory retrieval, repetitive self-focused processing may keep the search at an abstract and evaluative level, thereby impeding progression toward a temporally and contextually specific event. Prospective adolescent research has likewise linked rumination and executive control to later overgeneral autobiographical memory (Stewart et al., 2018).

The present network further suggests that Symptom Rumination was embedded beyond the ruminative-response community. Its BEI was second only to Experiential Avoidance, indicating substantial signed connectivity with other theoretical domains. This result is consistent with prior evidence linking repetitive negative thinking to autobiographical-memory overgenerality (L.-X. Cui & Huang, 2007) and childhood trauma to rumination-related psychological risk (Y. Cui et al., 2019). At the same time, the cross-sectional network cannot determine whether rumination precedes OGM, is maintained by overgeneral retrieval, or co-occurs with both because of shared affective or developmental processes.

Intimidation also showed high Expected Influence and was not part of a Goldbricker redundancy component. Its prominence is therefore less likely to reflect overlap with highly similar within-scale nodes. This finding is compatible with work linking emotional abuse and autobiographical-memory vulnerability in adolescence (Stange et al., 2013), but it should not be interpreted as showing that intimidation directly produces OGM. Repeated threat, criticism, or humiliation may shape emotion regulation, self-referential processing, and sensitivity to interpersonal danger, any of which could contribute to the wider pattern of conditional associations.

Avoidance of Threatening Stimuli and Contextual Elaboration also ranked highly on EI in the complete 20-node network. However, both belonged to Goldbricker-identified redundancy components. Their individual values should therefore be interpreted more cautiously than those of nodes that remained structurally distinct. The reduced-network results indicate that collapsing these overlapping components had very little effect on the rankings of unaffected nodes, which supports the broader network conclusions while cautioning against treating small rank differences among highly correlated subdimensions as evidence of unique causal importance. More generally, Strength and EI quantify structural prominence and do not directly measure symptom severity, adaptive value, causal influence, or intervention efficacy (Bringmann et al., 2019; Castro et al., 2024).

### 4.2. Bridge Nodes: Experiential Avoidance, Rumination, and Executive-Control Difficulties

Bridge Expected Influence provided a complementary view of cross-community connectivity (Jones et al., 2021). Experiential Avoidance had the highest BEI, followed by Symptom Rumination and Working Memory Difficulties. However, an important structural qualification is required. Experiential Avoidance was the sole node in its predefined community; consequently, every edge incident on this node was a cross-community edge, and its EI and BEI were identical by construction. Its high BEI therefore reflects broad connectivity with the remainder of the network rather than preferential connectivity with OGM.

The complementary OGM-specific analysis further differentiated these patterns. Working Memory Difficulties showed the greatest absolute conditional connectivity with the four OGM nodes (0.065), followed closely by Symptom Rumination (0.058), whereas Experiential Avoidance showed only minimal direct conditional connectivity with OGM (0.009). Thus, the present findings are more appropriately described as broadly compatible with the CaR-FA-X framework (Williams, 2006) than as demonstrating that functional avoidance constitutes the strongest bridge to OGM. Experiential avoidance appears structurally relevant to the broader cognitive–affective network, but its specific conditional association with the OGM community was comparatively weak. Prior work has likewise highlighted affect regulation and avoidance as relevant to autobiographical-memory specificity (Chen et al., 2012; Tian et al., 2018).

Symptom Rumination combined high within-network influence with strong cross-community connectivity. This convergence is consistent with a capture-and-rumination process that is closely coupled with avoidance and executive-control difficulties rather than functioning in isolation. Working Memory Difficulties showed the third-highest BEI. Interpretation of this result depends on the direction of ADEXI scoring: higher scores indicate greater working memory difficulty rather than better working-memory performance (Holst & Thorell, 2018). Thus, the finding is most appropriately read as greater working memory difficulty occupying a bridging position across communities. Difficulty maintaining a retrieval goal and filtering competing information is compatible with the executive-control component of CaR-FA-X (Williams, 2006).

Even with the improved stability evidence, BEI should not be interpreted as mediation or temporal transmission. Bridge centrality is a structural property of the estimated cross-sectional network and depends in part on the predefined community structure (Jones et al., 2021). The present results therefore identify robust candidate interfaces among domains, not established causal pathways.

### 4.3. Redundancy, Predictability, and Robustness of the Network Findings

The redundancy analysis identified five potentially redundant node pairs, all of which occurred within the predefined theoretical communities. These pairs formed three connected components: Spatiotemporal Construction–Contextual Elaboration, Brooding–Reflective Pondering, and a Cognitive-Avoidance component comprising Thought Substitution, Transformation of Images into Thoughts, Avoidance of Threatening Stimuli, and Thought Suppression. Notably, Distraction was not included in the Cognitive Avoidance redundancy component in the present dataset. Rather than deleting individual nodes after observing the network results, the primary analysis retained all 20 theory-defined nodes and evaluated a separate PC1-reduced sensitivity network. The resulting 15-node sensitivity network closely reproduced the primary findings. Among unchanged nodes, common-node edge weights were nearly identical across the primary and reduced networks, and the Jaccard overlap of nonzero edges was high. Node rankings were also strongly preserved, with rank correlations of 0.951 for Strength, 0.979 for Expected Influence, 0.993 for Bridge Expected Influence, and 1.000 for predictability. These findings reduce concern that the prominence of the principal nodes was primarily an artifact of topological overlap, while still supporting caution when interpreting small differences among redundancy-flagged subdimensions. Predictability provided information that centrality alone cannot supply. The average R^2^ of 0.679 indicates that, within the present sample, a substantial proportion of node variance was statistically accounted for by the other measured nodes. Symptom Rumination and several Cognitive Avoidance nodes had particularly high R^2^ values, whereas Indulgence was much less predictable. High predictability should not, however, be equated with causal controllability. A node can be well accounted for by its neighbors and still be influenced by unmeasured variables or common causes; similarly, a highly central node need not be the node whose manipulation produces the largest clinical change (Bringmann et al., 2019; Castro et al., 2024).

### 4.4. Gender Differences in Network Connectivity

The gender comparison provided stronger evidence for overall similarity than for systematic differences in network organization. The Network Comparison Test evaluates differences in estimated network structure and global strength (van Borkulo et al., 2023). In the primary NPN analysis, neither the overall network-structure test (M = 0.151, *p* = 0.166) nor the global-strength test (S = 0.238, *p* = 0.314) was statistically significant. Although estimated global strength was numerically higher in males than females, this difference was not statistically reliable. Moreover, none of the 190 edge-specific comparisons survived FDR correction. These findings therefore do not support the conclusion that male and female students possess fundamentally different network structures or consistently different levels of overall network connectivity.

Several exploratory FDR-adjusted node-centrality differences were observed. Interference showed higher Strength in males, while Expected Influence was higher in males for Interference and Indulgence and higher in females for Experiential Avoidance. These isolated differences should receive less interpretive weight than the nonsignificant overall network-structure and global-strength tests and the absence of FDR-significant edge differences. The Spearman sensitivity analysis similarly showed no significant network-structure difference and no FDR-significant edge differences, although it yielded a modest global-strength difference favoring males. Because this latter finding was not reproduced by the prespecified primary NPN analysis, evidence for a gender difference in overall connectivity should be regarded as estimation-dependent rather than robust.

The supplementary psychometric analyses provide some reassurance regarding measurement comparability across gender while also supporting a cautious interpretation of the gender network findings. For the OGMQ, RRS, experiential-avoidance, cognitive-avoidance, and ADEXI measures, increasingly restrictive equality constraints on factor loadings and intercepts produced only minimal changes in approximate fit, although absolute model fit was not uniformly strong. The complete invariance sequence could not be established for the childhood psychological abuse measure because some multigroup models did not converge. Nevertheless, node-level internal consistency was generally satisfactory in both gender groups. Taken together, these findings support retaining the gender network comparison as a secondary and exploratory analysis. Greater interpretive weight should therefore be placed on the overall network-structure test and FDR-corrected edge results than on isolated node-level or global-strength differences.

### 4.5. Theoretical and Practical Implications

Although the present pattern is compatible with CaR-FA-X, it should not be regarded as a unique confirmation of that model. Developmental psychopathology and latent-vulnerability perspectives propose that childhood maltreatment can become biologically and cognitively embedded through alterations in multiple neurocognitive systems, including emotion regulation and executive control (Gerin et al., 2019; McCrory et al., 2017). Such alterations may represent adaptations to adverse environments while simultaneously increasing vulnerability to later psychopathology. Emotion-regulation and cognitive-vulnerability perspectives likewise predict that childhood psychological abuse may become linked with later cognitive and emotional difficulties through multiple interacting processes. Prior studies have connected trauma-related reductions in autobiographical-memory specificity with impaired executive control and affect regulation (Chen et al., 2012), linked childhood trauma with broader emotion-regulation difficulties (Rashid et al., 2024), and showed a cognitive vulnerability–stress interaction involving emotional abuse and OGM in adolescence (Stange et al., 2013). These perspectives are not mutually exclusive with CaR-FA-X; rather, they suggest that rumination, avoidance, executive-control difficulties, and autobiographical-memory processes may be embedded within a broader developmental system.

This broader interpretation is also consistent with the sparse direct abuse–OGM edges in the primary network. Childhood psychological abuse was strongly organized within its own community, but its direct partial associations with OGM were small after conditioning on the remaining nodes. Such a pattern is compatible with the possibility that the statistical relationship between adversity and autobiographical-memory functioning is distributed across multiple affective and cognitive processes rather than represented by a single dominant direct pathway. The present cross-sectional design cannot determine whether these processes are mediators, consequences, or correlates.

The findings have potential implications for future school-based intervention research, but they do not identify established treatment targets. Centrality and bridge centrality should not be equated with causal leverage (Bringmann et al., 2019; Castro et al., 2024), and stronger evidence would require temporal or intervention designs relating node-specific change to subsequent changes elsewhere in the system (Blanken et al., 2019). Nevertheless, Symptom Rumination and Experiential Avoidance emerge as particularly plausible constructs for prospective evaluation because they were prominent across multiple analytic specifications, while Working Memory difficulties showed stable cross-community connectivity.

Two existing approaches may be especially relevant for future trials. Memory Specificity Training has improved autobiographical-memory specificity in depressed adolescents (Neshat-Doost et al., 2013), although its effects cannot be assumed to generalize directly to Chinese vocational high school students. Acceptance and Commitment Therapy, which aims to increase psychological flexibility and reduce rigid experiential avoidance, has also shown beneficial effects on stress-related outcomes in children and adolescents in a systematic review and meta-analysis of randomized trials (Binder et al., 2024). These approaches could be adapted for low-intensity school-based programs, but their effectiveness for OGM or the present network domains would need to be established prospectively.

The executive-function finding suggests a somewhat different practical direction. Because ADEXI scores reflect difficulty, not ability (Holst & Thorell, 2018), the Working Memory Difficulties result should not be taken as evidence that generic cognitive training would necessarily reduce OGM. School support might instead reduce everyday executive demands through structured planning, external reminders, manageable task sequencing, and reduced simultaneous task load. Whether such supports alter autobiographical-memory specificity remains an empirical question.

### 4.6. Limitations and Future Directions

Several limitations should be considered. First, the study was cross-sectional. The network describes conditional associations at a single time point and cannot establish temporal order, mediation, or causal influence. Longitudinal panel studies, ecological momentary assessment, and randomized interventions are needed to determine whether changes in rumination, experiential avoidance, executive-control difficulties, or autobiographical-memory processes precede changes elsewhere in the network. Network intervention analysis provides one possible framework for evaluating whether experimentally induced change in one node is followed by change in other nodes (Blanken et al., 2019).

Second, all constructs were assessed using self-report questionnaires. Shared response tendencies, overlapping item wording, and common method variance may therefore have contributed to some associations. This issue is especially relevant for executive functioning and autobiographical memory, for which questionnaire-based difficulty scores do not necessarily correspond directly to performance on behavioral tasks. Future work should combine self-report measures with performance-based autobiographical-memory tasks, behavioral measures of executive functioning, and, where feasible, parent or teacher reports. Although internal consistency was generally acceptable to excellent across the 20 network nodes, reliability was not identical across variables or gender groups. Measurement error can attenuate or otherwise influence estimated partial associations and may consequently affect Strength, Expected Influence, and Bridge Expected Influence rankings. Moreover, the supplementary confirmatory factor analyses showed mixed global fit across the source instruments, and complete gender measurement invariance could not be established for the childhood psychological abuse measure. These findings reinforce the need to interpret small differences in node rankings and gender-specific network estimates cautiously and to replicate the network using independent samples and complementary measurement approaches.

Third, the sensitivity analyses demonstrated substantial overlap among several within-scale subdimensions. The Goldbricker-reduced sensitivity analysis showed that the main conclusions were highly robust, but neither retaining all subscales nor replacing them with PC1 composites is uniquely definitive. Retaining subscales preserves theoretically meaningful distinctions, whereas PCA reduction sacrifices some construct-specific information in order to reduce topological overlap. Future studies using item-level network models, confirmatory measurement models, or independent samples could help determine the most appropriate level of aggregation.

Fourth, node predictability was estimated in the same sample used to estimate the network. The R^2^ values therefore quantify within-sample statistical predictability conditional on the variables included in this network and should not be interpreted as out-of-sample forecasting. Low predictability may reflect unmeasured influences rather than poor measurement, whereas high predictability can partly reflect shared construct variance.

Fifth, Bridge Expected Influence depends on the predefined community structure (Jones et al., 2021). The six communities were specified a priori on theoretical and measurement grounds, which makes the analysis interpretable but does not establish that this is the only valid community organization. Although BEI showed high case-dropping stability and was robust to redundancy reduction, alternative community specifications may yield somewhat different bridge estimates.

Finally, participants were recruited from vocational schools in Tianjin, Hebei, and Shandong, and the gender groups were unequal in size (822 male and 542 female students). The extent to which the present network generalizes to other regions, cultural contexts, school types, vocational-education pathways, or more gender-balanced samples therefore remains uncertain. Replication in five-year vocational programs, technical schools, academic high schools, and other educational systems would help determine which aspects of the network are population-specific and which are more generalizable.

In addition, the present network was deliberately restricted to variables closely related to the CaR-FA-X framework and therefore did not capture the full range of developmental and behavioral factors that may shape adolescent cognitive–affective functioning. For example, psychomotor agitation and related behavioral dysregulation may vary as a function of developmental and contextual factors such as chronotype and sleep-related processes (Figueiredo & Vieira, 2022). Future studies could examine whether incorporating such broader behavioral and developmental factors changes the organization of the abuse–OGM network.

## 5. Conclusions

This study used network analysis to examine the interrelations among childhood psychological abuse, overgeneralized autobiographical memory, and the processes described by the CaR-FA-X model in vocational high school students. Symptom Rumination showed the highest Expected Influence, whereas Experiential Avoidance, Symptom Rumination, and the Working Memory Difficulties node played the strongest cross-community bridging roles. Because higher ADEXI scores indicate greater difficulty, the Working Memory Difficulties finding reflects working memory difficulties rather than stronger working-memory ability. The male and female networks were broadly similar in structure, and the primary NPN analysis did not identify a significant difference in global strength; the modest difference observed in the Spearman sensitivity analysis was not sufficiently robust to support a general conclusion of stronger connectivity in males. Overall, rumination, experiential avoidance, and executive-control difficulties were closely embedded in the broader network linking childhood psychological abuse with autobiographical memory. Given the cross-sectional design, central and bridge nodes should be regarded as theoretically and clinically relevant correlates rather than established causal mechanisms or intervention targets. Longitudinal and experimental studies are needed to determine whether changes in these processes precede or contribute to subsequent changes in autobiographical-memory specificity and other components of the network.

## Figures and Tables

**Figure 1:**
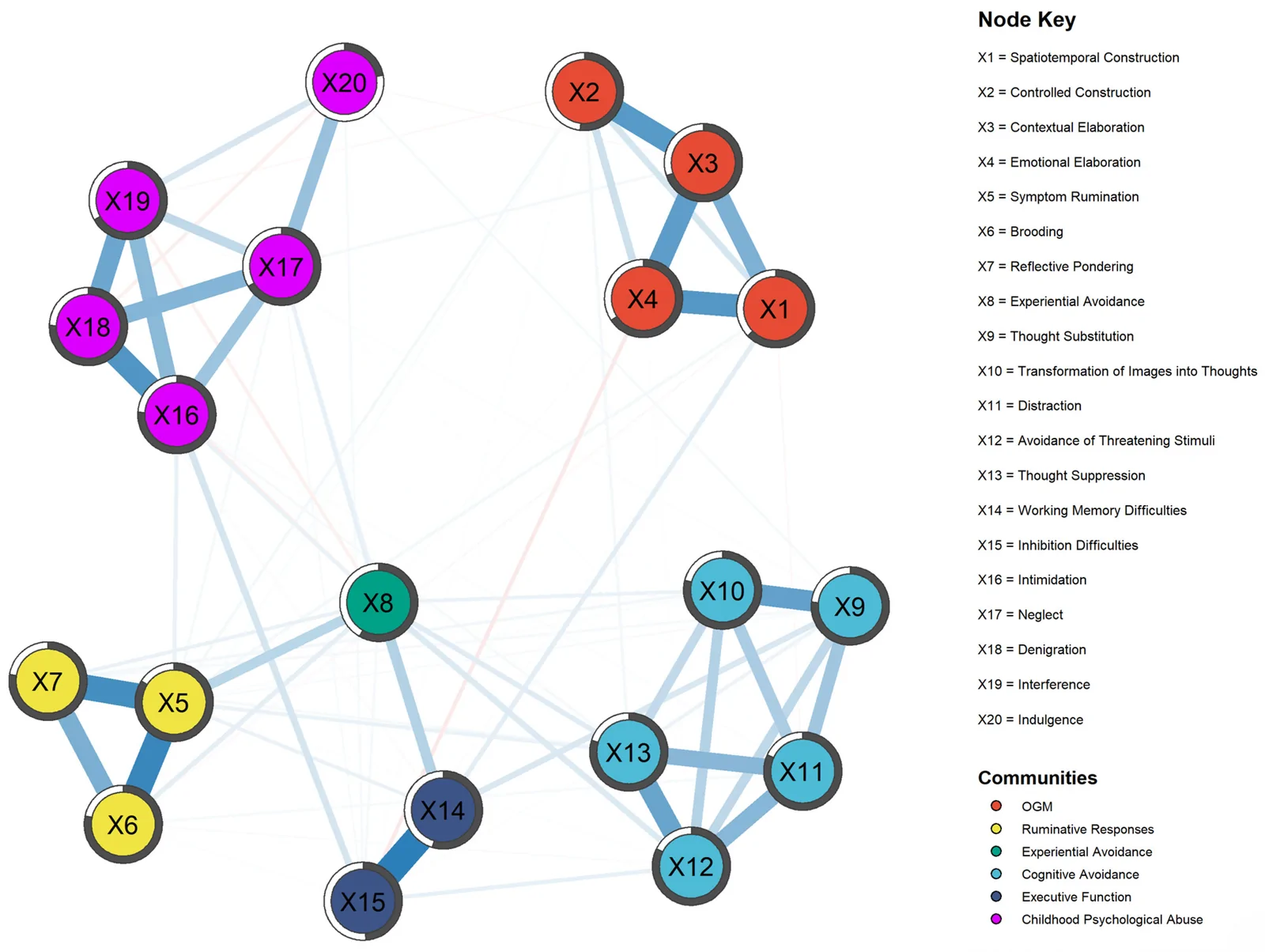
Primary 20-node NPN-EBICglasso network with node predictability rings. Blue edges indicate positive regularized partial correlations and red edges indicate negative regularized partial correlations; thicker edges indicate larger absolute edge weights. Node colors denote the six a priori communities. The outer ring represents within-sample node predictability (R^2^), with a larger filled proportion indicating that more variance in the node is statistically accounted for by its neighboring nodes.

**Figure 2:**
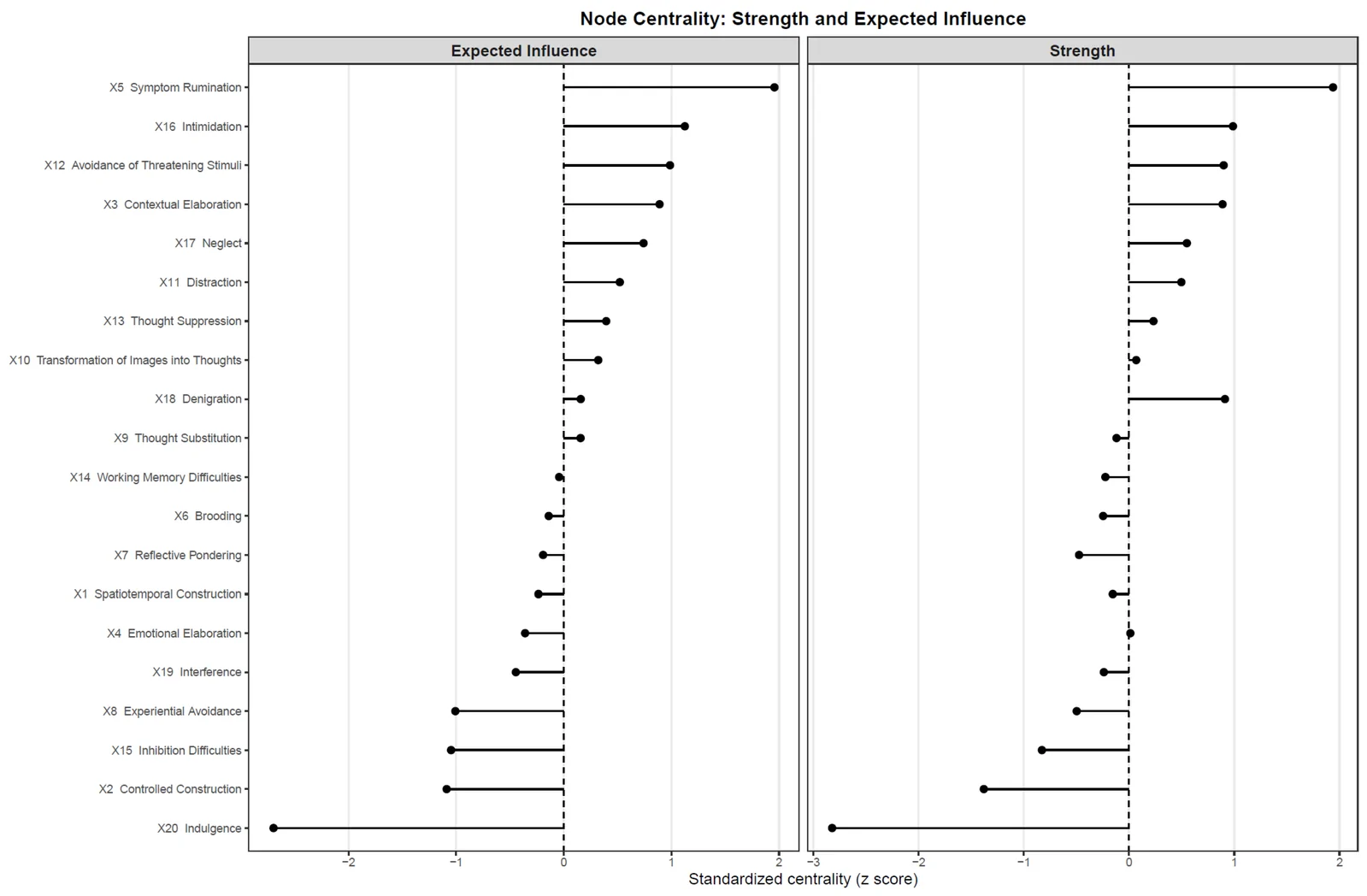
Standardized Strength and Expected Influence for the 20 nodes. Each centrality metric was standardized separately as a z score.

**Figure 3:**
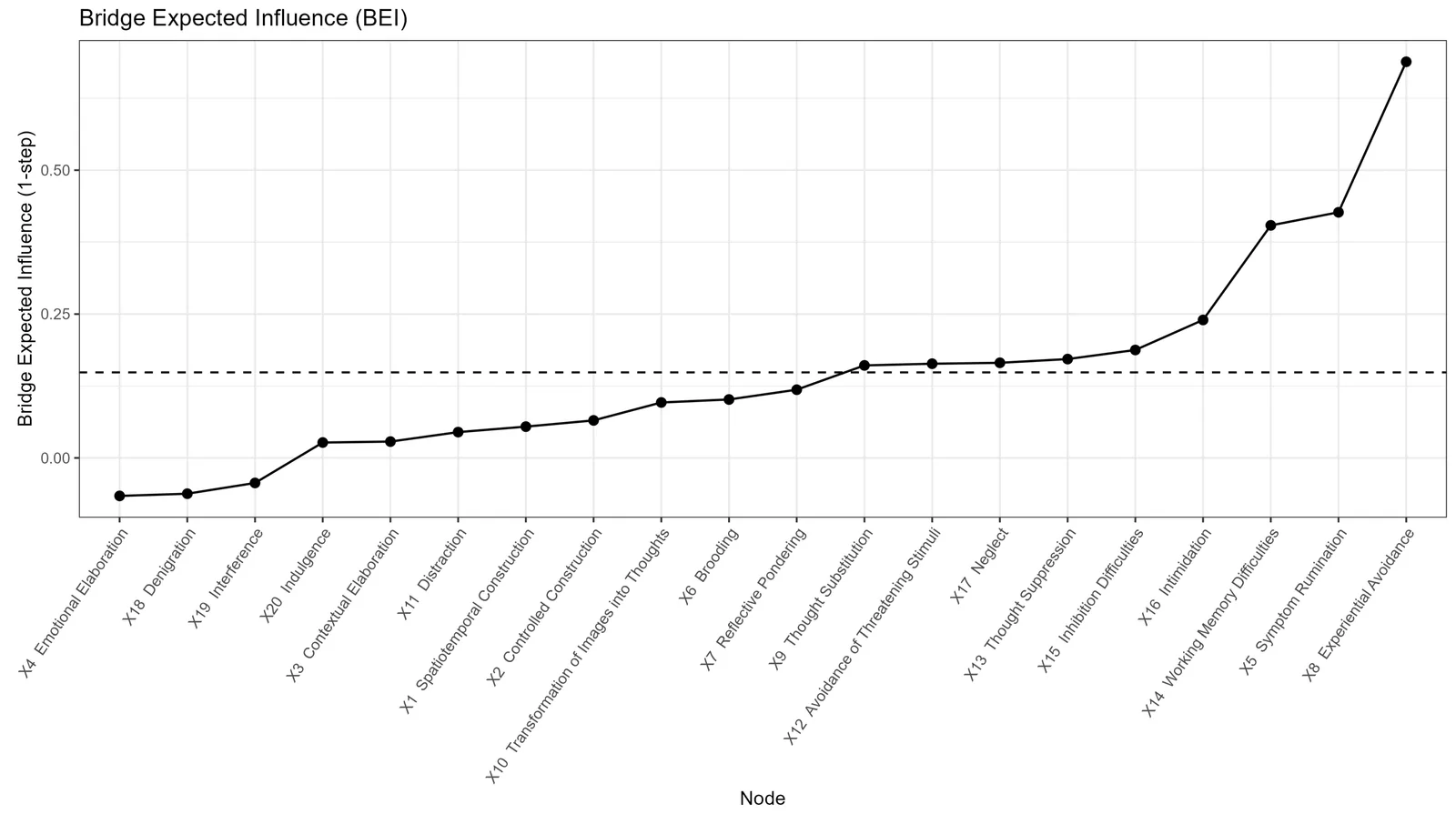
One-step Bridge Expected Influence for the 20 nodes. The dashed horizontal line indicates the mean BEI across nodes.

**Figure 4:**
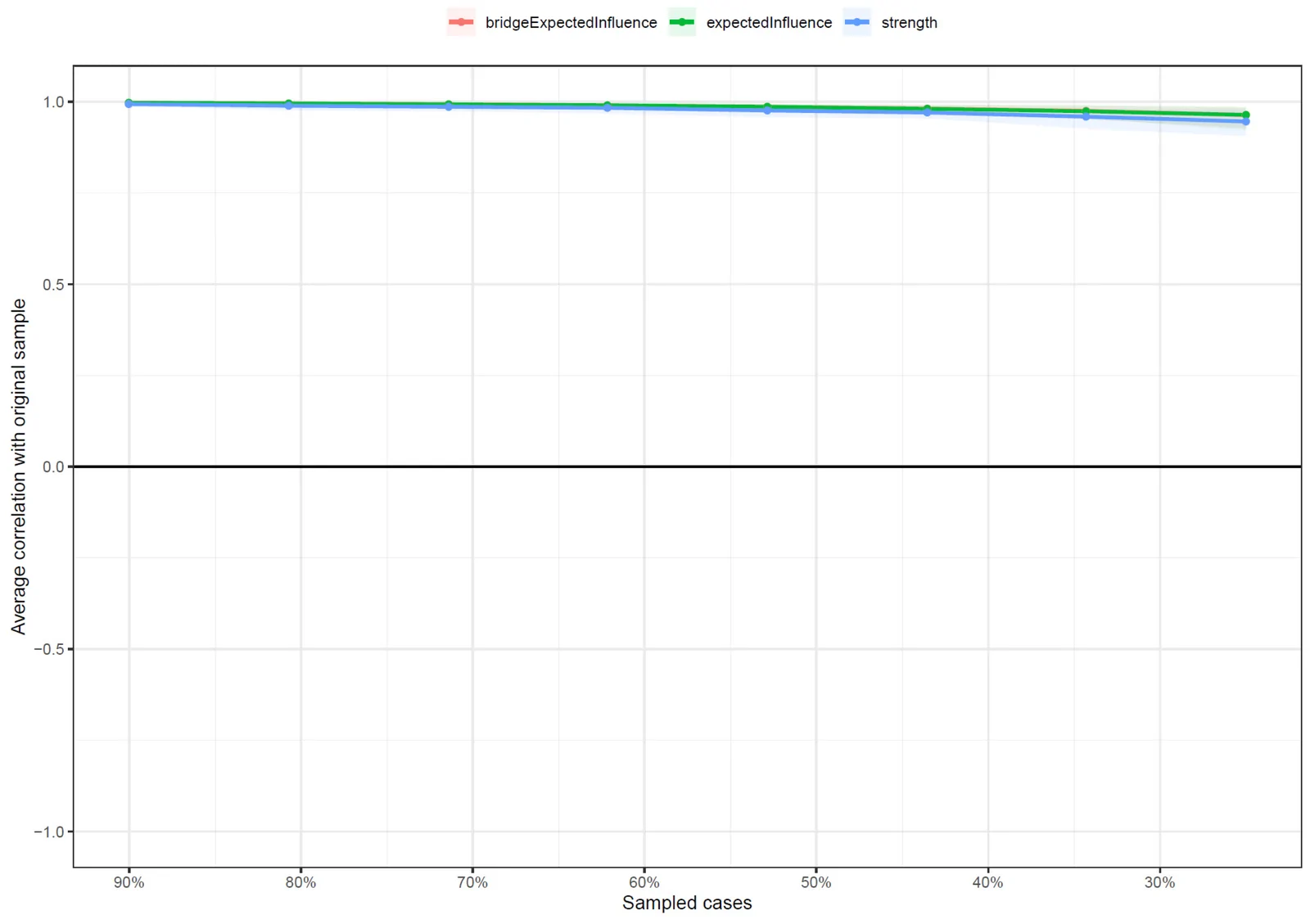
Case-dropping bootstrap stability of Strength, Expected Influence, and Bridge Expected Influence. Lines represent the average correlation between subset estimates and the original full-sample estimates across progressively smaller sampled proportions.

**Table 1:** Descriptive statistics for the 20 study variables.

Node	Variable	M	SD	Median	Skewness	Kurtosis
X1	Spatiotemporal Construction	8.87	3.66	8	1.15	1.02
X2	Controlled Construction	6.13	2.45	6	0.62	−0.20
X3	Contextual Elaboration	11.76	4.19	12	0.69	0.34
X4	Emotional Elaboration	8.87	3.56	8	1.12	1.09
X5	Symptom Rumination	22.93	8.08	23	0.64	0.22
X6	Brooding	10.38	4	10	0.48	−0.43
X7	Reflective Pondering	9.48	3.62	9	0.73	0.15
X8	Experiential Avoidance	19.56	10.56	18	0.62	−0.35
X9	Thought Substitution	10.49	4.81	10	0.92	0.6
X10	Transformation of Images into Thoughts	10.99	5.28	10	0.76	−0.15
X11	Distraction	11.52	5.34	11	0.65	−0.23
X12	Avoidance of Threatening Stimuli	11.7	5.59	11	0.66	−0.37
X13	Thought Suppression	12.02	5.48	11	0.66	−0.26
X14	Working Memory Difficulties	24	9.14	25	−0.01	−0.68
X15	Inhibition Difficulties	16.19	5.16	17	−0.65	−0.17
X16	Intimidation	7.62	6.77	7	0.76	−0.05
X17	Neglect	5.49	5.58	4	1.04	0.49
X18	Denigration	3.53	4.02	2	1.1	0.48
X19	Interference	3.57	3.93	2	1.07	0.36
X20	Indulgence	2.13	2.7	1	1.02	−0.29

*Note. N = 1364 for all variables. Higher scores on Working Memory Difficulties and Inhibition Difficulties indicate greater executive-function difficulties.*

**Table 2:** Node Strength, Expected Influence, Bridge Expected Influence, and predictability.

Node	Variable	Community	Strength	EI	BEI	R^2^
X1	Spatiotemporal Construction	OGM	0.933	0.855	0.054	0.626
X2	Controlled Construction	OGM	0.701	0.671	0.065	0.519
X3	Contextual Elaboration	OGM	1.13	1.098	0.028	0.696
X4	Emotional Elaboration	OGM	0.964	0.828	−0.066	0.656
X5	Symptom Rumination	Ruminative Responses	1.328	1.328	0.427	0.841
X6	Brooding	Ruminative Responses	0.915	0.875	0.102	0.784
X7	Reflective Pondering	Ruminative Responses	0.872	0.864	0.119	0.777
X8	Experiential Avoidance	Experiential Avoidance	0.868	0.688	0.688	0.583
X9	Thought Substitution	Cognitive Avoidance	0.939	0.939	0.161	0.766
X10	Transformation of Images into Thoughts	Cognitive Avoidance	0.975	0.975	0.096	0.791
X11	Distraction	Cognitive Avoidance	1.056	1.018	0.045	0.818
X12	Avoidance of Threatening Stimuli	Cognitive Avoidance	1.132	1.118	0.164	0.824
X13	Thought Suppression	Cognitive Avoidance	1.006	0.991	0.172	0.798
X14	Working Memory Difficulties	Executive Function	0.919	0.896	0.404	0.546
X15	Inhibition Difficulties	Executive Function	0.805	0.68	0.188	0.489
X16	Intimidation	Childhood Psychological Abuse	1.148	1.148	0.24	0.76
X17	Neglect	Childhood Psychological Abuse	1.066	1.066	0.165	0.664
X18	Denigration	Childhood Psychological Abuse	1.134	0.94	−0.062	0.754
X19	Interference	Childhood Psychological Abuse	0.917	0.81	−0.044	0.667
X20	Indulgence	Childhood Psychological Abuse	0.429	0.324	0.027	0.221

*Note. EI = one-step Expected Influence; BEI = one-step Bridge Expected Influence. R^2^ is the within-sample node predictability estimate.*

**Table 3:** Goldbricker-connected redundancy components and PCA reduction.

Composite	Community	Source Nodes	Source Variables	PC1 Variance Explained
RC1	OGM	X1, X3	Spatiotemporal Construction; Contextual Elaboration	86.7%
RC2	Ruminative Responses	X6, X7	Brooding; Reflective Pondering	90.8%
RC3	Cognitive Avoidance	X9, X10, X12, X13	Thought Substitution; Transformation of Images into Thoughts; Avoidance of Threatening Stimuli; Thought Suppression	84.9%

*Note. Connected Goldbricker components were reduced only within the same predefined community. The primary 20-node network remained unchanged.*

**Table 4:** Bootstrap stability and sensitivity-analysis summary.

Analysis	Metric	Estimate
Case-dropping	CS—Strength	0.750
	CS—Expected Influence	0.750
	CS—Bridge Expected Influence	0.750
NPN vs. Spearman	Pearson correlation of all edge weights	0.998
	Spearman correlation of all edge weights	0.963
	Jaccard overlap of nonzero edges	0.868
	Rank correlation—Strength	0.979
	Rank correlation—Expected Influence	0.995
	Rank correlation—Bridge Expected Influence	0.991
Primary vs. reduced	Pearson correlation of common-node edge weights	0.999
	Spearman correlation of common-node edge weights	0.979
	Jaccard overlap of nonzero common-node edges	0.923
	Rank correlation—Strength	0.951
	Rank correlation—Expected Influence	0.979
	Rank correlation—Bridge Expected Influence	0.993
	Rank correlation—predictability R^2^	1.000

*Note. Rank correlations are Spearman correlations. Primary-versus-reduced node-level comparisons are restricted to unchanged original nodes; PC1 composites are not treated as equivalent to any single source node.*

**Table 5:** Network Comparison Test results by gender.

Estimation	Test/Quantity	Male	Female	Statistic	*p*/q
Primary NPN	Network structure			M = 0.151	*p* = 0.166
Primary NPN	Global strength	9.313	9.075	S = 0.238	*p* = 0.314
Primary NPN	FDR-significant edges	0/190			min q = 0.295
Spearman sensitivity	Network structure	—	—	M = 0.145	*p* = 0.219
Spearman sensitivity	Global strength	9.392	8.89	S = 0.502	*p* = 0.044
Spearman sensitivity	FDR-significant edges	0 of 190			min q = 0.391

*Note. M = Network Invariance Test statistic; S = Global Strength Invariance Test statistic. Edge-specific p values were adjusted using the false discovery rate.*

## Data Availability

The raw data supporting the conclusions of this article will be made available by the authors on request.

## Supplementary Materials

The following supporting information can be downloaded at https://www.mdpi.com/article/10.3390/bs16101773/s1. Table S1, Zero-order Spearman correlation matrix for the 20 network nodes; Table S2, Node-level reliability estimates for the 20 network variables in the full sample and by gender; Table S3, Gender-specific descriptive statistics for the 20 network variables; Table S4, Confirmatory factor analysis fit indices for the six source measures; Table S5, Standardized CFA factor loadings for the six source measures; Table S6, Gender measurement invariance results for the six source measures; Table S7, OGM-specific conditional edges and connectivity of CaR-FA-X-related processes; Table S8, Goldbricker-identified redundancy candidate pairs; Table S9, FDR-adjusted node-centrality differences between male and female networks in the primary NPN analysis; Figure S1, Spearman sensitivity analyses (Panels A–B); Figure S2, Nonparametric bootstrap 95% confidence intervals for edge weights; Figure S3, Bootstrap difference tests (Panels A–C); Figure S4, Goldbricker-reduced sensitivity analyses (Panels A–D); Figure S5, Gender-specific primary NPN networks (Panels A–B); Figure S6, OGM-specific conditional connectivity in the primary NPN network; Supplementary Data S1, Full FDR-adjusted edge-specific Network Comparison Test results.
