# Supplementary material for: A Network Analysis of Childhood Psychological Abuse and Overgeneralized Autobiographical Memory in Vocational High School Students
Source: Behav Sci (Basel). 2026 Sep 28;16(10):1773. doi: 10.3390/bs16101773 (PMC13701399; doi:10.3390/bs16101773)
Supplement: Supplementary file 1 [file behavsci-16-01773-s001.zip › Supplementary_Materials_N1364.pdf]

Supplementary Materials

A Network Analysis of Childhood Psychological Abuse and Overgeneralized Autobiographical Memory in Vocational High School Students

Final corrected analytic sample: N = 1,364 (822 male, 542 female)

Supplementary tables and figures corresponding to the final corrected psychometric and network analyses.

Contents

- Table S1. Zero-order Spearman correlation matrix for the 20 network nodes.
- Table S2. Node-level reliability estimates for the 20 network variables in the full sample and by gender.
- Table S3. Gender-specific descriptive statistics for the 20 network variables.
- Table S4. Confirmatory factor analysis fit indices for the six source measures.
- Table S5. Standardized CFA factor loadings for the six source measures.
- Table S6. Gender measurement invariance results for the six source measures.
- Table S7. OGM-specific conditional edges and connectivity of CaR-FA-X-related processes.
- Table S8. Goldbricker-identified redundancy candidate pairs.
- Table S9. FDR-adjusted node-centrality differences between male and female networks in the primary NPN analysis.
- Figure S1. Spearman sensitivity analyses (Panels A–B).
- Figure S2. Nonparametric bootstrap 95% confidence intervals for edge weights.
- Figure S3. Bootstrap difference tests (Panels A–C).
- Figure S4. Goldbricker-reduced sensitivity analyses (Panels A–D).
- Figure S5. Gender-specific primary NPN networks (Panels A–B).
- Figure S6. OGM-specific conditional connectivity in the primary NPN network.
- Supplementary Data S1. Full FDR-adjusted edge-specific Network Comparison Test results.

Table S1. Zero-Order Spearman Correlation Matrix for the 20 Network Nodes

Node	X1	X2	X3	X4	X5	X6	X7	X8	X9	X10	X11	X12	X13	X14	X15	X16	X17	X18	X19	X20
X1	1.000	.561	.694	.707	.190	.119	.155	.139	.126	.121	.089	.109	.126	.190	.051	.078	.115	.074	.055	.027
X2	.561	1.000	.669	.573	.232	.216	.178	.180	.145	.164	.153	.188	.201	.179	.113	.111	.122	.076	.052	.026
X3	.694	.669	1.000	.724	.192	.155	.137	.119	.130	.139	.126	.135	.152	.170	.087	.109	.139	.095	.088	.008
X4	.707	.573	.724	1.000	.134	.094	.108	.072	.072	.064	.051	.059	.071	.090	-.001	.052	.095	.043	.040	-.001
X5	.190	.232	.192	.134	1.000	.857	.852	.696	.605	.621	.624	.635	.640	.505	.391	.443	.423	.329	.313	.192
X6	.119	.216	.155	.094	.857	1.000	.809	.636	.551	.576	.585	.590	.589	.450	.373	.392	.375	.290	.273	.187
X7	.155	.178	.137	.108	.852	.809	1.000	.641	.573	.594	.594	.589	.590	.443	.327	.379	.376	.294	.290	.177
X8	.139	.180	.119	.072	.696	.636	.641	1.000	.579	.607	.609	.629	.627	.536	.415	.398	.389	.269	.253	.171
X9	.126	.145	.130	.072	.605	.551	.573	.579	1.000	.828	.804	.802	.761	.498	.376	.385	.362	.304	.299	.214
X10	.121	.164	.139	.064	.621	.576	.594	.607	.828	1.000	.820	.823	.801	.478	.372	.390	.364	.286	.288	.201
X11	.089	.153	.126	.051	.624	.585	.594	.609	.804	.820	1.000	.854	.838	.465	.397	.398	.372	.295	.290	.192
X12	.109	.188	.135	.059	.635	.590	.589	.629	.802	.823	.854	1.000	.847	.494	.425	.403	.369	.293	.288	.186
X13	.126	.201	.152	.071	.640	.589	.590	.627	.761	.801	.838	.847	1.000	.461	.403	.399	.369	.282	.277	.192
X14	.190	.179	.170	.090	.505	.450	.443	.536	.498	.478	.465	.494	.461	1.000	.618	.343	.353	.247	.236	.160
X15	.051	.113	.087	-.001	.391	.373	.327	.415	.376	.372	.397	.425	.403	.618	1.000	.396	.349	.293	.298	.205
X16	.078	.111	.109	.052	.443	.392	.379	.398	.385	.390	.398	.403	.399	.343	.396	1.000	.756	.823	.770	.377
X17	.115	.122	.139	.095	.423	.375	.376	.389	.362	.364	.372	.369	.369	.353	.349	.756	1.000	.751	.694	.460
X18	.074	.076	.095	.043	.329	.290	.294	.269	.304	.286	.295	.293	.282	.247	.293	.823	.751	1.000	.784	.325
X19	.055	.052	.088	.040	.313	.273	.290	.253	.299	.288	.290	.288	.277	.236	.298	.770	.694	.784	1.000	.388
X20	.027	.026	.008	-.001	.192	.187	.177	.171	.214	.201	.192	.186	.192	.160	.205	.377	.460	.325	.388	1.000

Note. Values are Spearman rank-order correlations. Node labels are given below.

Node	Variable	Community
X1	Spatiotemporal Construction	OGM
X2	Controlled Construction	OGM
X3	Contextual Elaboration	OGM
X4	Emotional Elaboration	OGM
X5	Symptom Rumination	Ruminative Responses
X6	Brooding	Ruminative Responses
X7	Reflective Pondering	Ruminative Responses
X8	Experiential Avoidance	Experiential Avoidance
X9	Thought Substitution	Cognitive Avoidance
X10	Transformation of Images into Thoughts	Cognitive Avoidance
X11	Distraction	Cognitive Avoidance
X12	Avoidance of Threatening Stimuli	Cognitive Avoidance
X13	Thought Suppression	Cognitive Avoidance
X14	Working Memory Difficulties	Executive Function
X15	Inhibition Difficulties	Executive Function
X16	Intimidation	Childhood Psychological Abuse
X17	Neglect	Childhood Psychological Abuse
X18	Denigration	Childhood Psychological Abuse
X19	Interference	Childhood Psychological Abuse
X20	Indulgence	Childhood Psychological Abuse

Table S2. Node-Level Reliability Estimates for the 20 Network Variables in the Full Sample and by Gender

Node	Variable	Items	Full α	Full ω/SB	Male α	Male ω/SB	Female α	Female ω/SB
X1	Spatiotemporal Construction	5	.806	.866	.842	.895	.714	.795
X2	Controlled Construction	3	.776	.833	.794	.852	.741	.796
X3	Contextual Elaboration	6	.777	.838	.801	.861	.730	.794
X4	Emotional Elaboration	5	.780	.845	.809	.870	.720	.792
X5	Symptom Rumination	12	.921	.946	.933	.956	.896	.924
X6	Brooding	5	.865	.904	.883	.921	.822	.862
X7	Reflective Pondering	5	.827	.878	.865	.910	.746	.805
X8	Experiential Avoidance	7	.927	.948	.934	.955	.908	.928
X9	Thought Substitution	5	.854	.897	.891	.927	.779	.834
X10	Transformation of Images into Thoughts	5	.884	.921	.913	.943	.829	.872
X11	Distraction	5	.884	.919	.905	.937	.838	.876
X12	Avoidance of Threatening Stimuli	5	.903	.933	.914	.943	.878	.908
X13	Thought Suppression	5	.888	.918	.903	.934	.854	.882
X14	Working Memory Difficulties	9	.911	.933	.933	.951	.858	.885
X15	Inhibition Difficulties	5	.818	.850	.838	.871	.739	.780
X16	Intimidation	7	.880	.919	.881	.923	.876	.910
X17	Neglect	6	.842	.903	.867	.922	.796	.863
X18	Denigration	4	.827	.894	.840	.910	.805	.866
X19	Interference	4	.808	.882	.826	.899	.776	.852
X20	Indulgence	2	.732	.734 (SB)	.714	.714 (SB)	.762	.768 (SB)

Note. α = Cronbach's alpha; ω = McDonald's omega total estimated from polychoric correlations. For the two-item Indulgence subscale (X20), the Spearman–Brown coefficient (SB) is reported instead of omega.

Table S3. Gender-Specific Descriptive Statistics for the 20 Network Variables

Node	Variable	Gender	N	Mean	SD	Median	Min	Max	Skewness	Kurtosis
X1	Spatiotemporal Construction	Male	822	9.11	3.96	8	5	20	1.06	.51
X1	Spatiotemporal Construction	Female	542	8.51	3.10	8	5	20	1.15	1.65
X2	Controlled Construction	Male	822	5.93	2.49	6	3	12	.73	-.09
X2	Controlled Construction	Female	542	6.44	2.35	6	3	12	.49	-.25
X3	Contextual Elaboration	Male	822	11.70	4.39	12	6	24	.74	.27
X3	Contextual Elaboration	Female	542	11.86	3.87	12	6	24	.59	.39
X4	Emotional Elaboration	Male	822	9.04	3.75	8	5	20	1.02	.69
X4	Emotional Elaboration	Female	542	8.61	3.23	8	5	20	1.25	1.80
X5	Symptom Rumination	Male	822	21.86	8.25	21	12	48	.86	.62
X5	Symptom Rumination	Female	542	24.55	7.55	24	12	48	.40	-.05
X6	Brooding	Male	822	9.74	4.00	10	5	20	.67	-.16
X6	Brooding	Female	542	11.34	3.80	11	5	20	.30	-.54
X7	Reflective Pondering	Male	822	9.12	3.77	9	5	20	.91	.35
X7	Reflective Pondering	Female	542	10.03	3.30	10	5	20	.51	.04
X8	Experiential Avoidance	Male	822	17.74	10.37	15	7	49	.88	.15
X8	Experiential Avoidance	Female	542	22.32	10.25	22	7	49	.35	-.62
X9	Thought Substitution	Male	822	10.26	5.06	10	5	25	1.02	.66
X9	Thought Substitution	Female	542	10.84	4.40	10	5	25	.76	.48
X10	Transformation of Images into Thoughts	Male	822	10.46	5.41	10	5	25	.94	.14
X10	Transformation of Images into Thoughts	Female	542	11.78	4.98	11	5	25	.53	-.39
X11	Distraction	Male	822	10.74	5.39	10	5	25	.88	.15
X11	Distraction	Female	542	12.70	5.03	12	5	25	.41	-.43
X12	Avoidance of Threatening Stimuli	Male	822	10.84	5.49	10	5	25	.85	-.02
X12	Avoidance of Threatening Stimuli	Female	542	13.01	5.49	12	5	25	.45	-.60
X13	Thought Suppression	Male	822	11.29	5.53	10	5	25	.84	-.02
X13	Thought Suppression	Female	542	13.12	5.23	13	5	25	.49	-.37
X14	Working Memory Difficulties	Male	822	23.00	9.74	23	9	45	.17	-.77
X14	Working Memory Difficulties	Female	542	25.52	7.91	26	9	45	-.24	-.36
X15	Inhibition Difficulties	Male	822	15.26	5.45	16	5	25	-.43	-.56
X15	Inhibition Difficulties	Female	542	17.59	4.34	18	5	25	-.95	.95
X16	Intimidation	Male	822	6.93	6.68	6	0	28	.88	.23
X16	Intimidation	Female	542	8.65	6.79	8	0	28	.61	-.33
X17	Neglect	Male	822	5.17	5.79	4	0	24	1.20	.85
X17	Neglect	Female	542	5.96	5.20	5	0	24	.77	-.12

Node	Variable	Gender	N	Mean	SD	Median	Min	Max	Skewness	Kurtosis
X18	Denigration	Male	822	3.32	4.09	2	0	16	1.18	.62
X18	Denigration	Female	542	3.84	3.89	3	0	16	.98	.32
X19	Interference	Male	822	3.49	4.09	2	0	16	1.14	.47
X19	Interference	Female	542	3.70	3.66	3	0	16	.92	.05
X20	Indulgence	Male	822	2.11	2.66	1	0	8	1.03	-.23
X20	Indulgence	Female	542	2.16	2.76	0	0	8	1.00	-.38

Note. Male n = 822; female n = 542. Higher ADEXI Working Memory Difficulties and Inhibition Difficulties scores indicate greater executive-function difficulties.

Table S4. Confirmatory Factor Analysis Fit Indices for the Six Source Measures

Scale	Converged	χ^2	df	p	CFI	TLI	RMSEA	SRMR
OGMQ	Yes	1224.49	146	<.001	.901	.885	.090	.044
RRS	Yes	2365.65	206	<.001	.894	.881	.099	.041
AAQ-II	Yes	317.59	14	<.001	.965	.948	.125	.025
Cognitive Avoidance Questionnaire	Yes	3457.93	265	<.001	.914	.903	.091	.034
ADEXI	Yes	806.84	76	<.001	.951	.942	.076	.034
Child Psychological Abuse Scale	Yes	3914.44	220	<.001	.808	.780	.136	.065

Note. CFA models used the prespecified factor structures and WLSMV estimation for ordinal indicators. These analyses were treated as supplementary psychometric diagnostics rather than as a new validation study.

Table S5. Standardized CFA Factor Loadings for the Six Source Measures

Scale	Node	Factor / subscale	Item	Std. loading	SE	z	p
OGMQ	X1	Spatiotemporal Construction	OGMQ-1	.736	.018	41.01	<.001
OGMQ	X1	Spatiotemporal Construction	OGMQ-7	.680	.017	39.89	<.001
OGMQ	X1	Spatiotemporal Construction	OGMQ-8	.758	.015	52.15	<.001
OGMQ	X1	Spatiotemporal Construction	OGMQ-14	.768	.014	55.78	<.001
OGMQ	X1	Spatiotemporal Construction	OGMQ-16	.825	.012	69.87	<.001
OGMQ	X2	Controlled Construction	OGMQ-6	.771	.014	53.71	<.001
OGMQ	X2	Controlled Construction	OGMQ-11	.796	.012	63.69	<.001
OGMQ	X2	Controlled Construction	OGMQ-17	.809	.013	60.75	<.001
OGMQ	X3	Contextual Elaboration	OGMQ-3	.684	.015	44.62	<.001
OGMQ	X3	Contextual Elaboration	OGMQ-4	.510	.021	23.81	<.001
OGMQ	X3	Contextual Elaboration	OGMQ-9	.691	.016	42.65	<.001
OGMQ	X3	Contextual Elaboration	OGMQ-12	.695	.015	45.89	<.001
OGMQ	X3	Contextual Elaboration	OGMQ-13	.716	.014	49.61	<.001
OGMQ	X3	Contextual Elaboration	OGMQ-18	.784	.013	62.08	<.001
OGMQ	X4	Emotional Elaboration	OGMQ-2	.670	.019	35.71	<.001
OGMQ	X4	Emotional Elaboration	OGMQ-5	.709	.017	42.13	<.001
OGMQ	X4	Emotional Elaboration	OGMQ-10	.791	.013	62.09	<.001
OGMQ	X4	Emotional Elaboration	OGMQ-15	.764	.014	54.00	<.001
OGMQ	X4	Emotional Elaboration	OGMQ-19	.711	.016	45.24	<.001
RRS	X5	Symptom Rumination	RRS-1	.751	.014	54.93	<.001
RRS	X5	Symptom Rumination	RRS-2	.688	.015	45.40	<.001
RRS	X5	Symptom Rumination	RRS-3	.753	.013	60.17	<.001
RRS	X5	Symptom Rumination	RRS-4	.704	.014	49.01	<.001
RRS	X5	Symptom Rumination	RRS-6	.811	.011	76.88	<.001
RRS	X5	Symptom Rumination	RRS-8	.778	.012	67.61	<.001
RRS	X5	Symptom Rumination	RRS-9	.839	.009	93.75	<.001
RRS	X5	Symptom Rumination	RRS-14	.769	.012	63.79	<.001
RRS	X5	Symptom Rumination	RRS-17	.847	.009	97.82	<.001
RRS	X5	Symptom Rumination	RRS-18	.788	.011	69.67	<.001
RRS	X5	Symptom Rumination	RRS-19	.766	.012	61.71	<.001
RRS	X5	Symptom Rumination	RRS-22	.769	.012	61.84	<.001
RRS	X6	Brooding	RRS-5	.805	.011	75.04	<.001
RRS	X6	Brooding	RRS-10	.844	.009	95.14	<.001
RRS	X6	Brooding	RRS-13	.741	.013	55.38	<.001
RRS	X6	Brooding	RRS-15	.816	.010	82.87	<.001
RRS	X6	Brooding	RRS-16	.845	.009	97.92	<.001
RRS	X7	Reflective Pondering	RRS-7	.781	.011	68.66	<.001
RRS	X7	Reflective Pondering	RRS-11	.821	.011	78.13	<.001
RRS	X7	Reflective Pondering	RRS-12	.645	.018	36.35	<.001
RRS	X7	Reflective Pondering	RRS-20	.827	.010	85.34	<.001
RRS	X7	Reflective Pondering	RRS-21	.769	.012	62.38	<.001
AAQ-II	X8	Experiential Avoidance	AAQ-1	.833	.010	86.59	<.001

Scale	Node	Factor / subscale	Item	Std. loading	SE	z	p
AAQ-II	X8	Experiential Avoidance	AAQ-2	.858	.008	105.19	<.001
AAQ-II	X8	Experiential Avoidance	AAQ-3	.869	.008	109.94	<.001
AAQ-II	X8	Experiential Avoidance	AAQ-4	.869	.008	107.46	<.001
AAQ-II	X8	Experiential Avoidance	AAQ-5	.894	.007	135.71	<.001
AAQ-II	X8	Experiential Avoidance	AAQ-6	.842	.009	95.16	<.001
AAQ-II	X8	Experiential Avoidance	AAQ-7	.795	.011	71.71	<.001
Cognitive Avoidance Questionnaire	X9	Thought Substitution	RZHB-4	.773	.012	65.37	<.001
Cognitive Avoidance Questionnaire	X9	Thought Substitution	RZHB-11	.820	.010	82.64	<.001
Cognitive Avoidance Questionnaire	X9	Thought Substitution	RZHB-17	.829	.010	86.55	<.001
Cognitive Avoidance Questionnaire	X9	Thought Substitution	RZHB-20	.819	.010	80.75	<.001
Cognitive Avoidance Questionnaire	X9	Thought Substitution	RZHB-25	.745	.013	57.13	<.001
Cognitive Avoidance Questionnaire	X10	Transformation of Images into Thoughts	RZHB-3	.824	.010	80.38	<.001
Cognitive Avoidance Questionnaire	X10	Transformation of Images into Thoughts	RZHB-15	.850	.009	95.38	<.001
Cognitive Avoidance Questionnaire	X10	Transformation of Images into Thoughts	RZHB-19	.855	.009	99.94	<.001
Cognitive Avoidance Questionnaire	X10	Transformation of Images into Thoughts	RZHB-23	.830	.009	89.06	<.001
Cognitive Avoidance Questionnaire	X10	Transformation of Images into Thoughts	RZHB-24	.842	.009	95.28	<.001
Cognitive Avoidance Questionnaire	X11	Distraction	RZHB-8	.870	.008	112.07	<.001
Cognitive Avoidance Questionnaire	X11	Distraction	RZHB-10	.826	.009	89.78	<.001
Cognitive Avoidance Questionnaire	X11	Distraction	RZHB-12	.818	.009	87.63	<.001
Cognitive Avoidance Questionnaire	X11	Distraction	RZHB-13	.843	.008	100.15	<.001
Cognitive Avoidance Questionnaire	X11	Distraction	RZHB-21	.832	.009	89.04	<.001
Cognitive Avoidance Questionnaire	X12	Avoidance of Threatening Stimuli	RZHB-7	.854	.008	104.32	<.001
Cognitive Avoidance Questionnaire	X12	Avoidance of Threatening Stimuli	RZHB-9	.850	.008	100.32	<.001
Cognitive Avoidance Questionnaire	X12	Avoidance of Threatening Stimuli	RZHB-16	.859	.008	108.22	<.001
Cognitive Avoidance Questionnaire	X12	Avoidance of Threatening Stimuli	RZHB-18	.873	.007	117.06	<.001
Cognitive Avoidance Questionnaire	X12	Avoidance of Threatening Stimuli	RZHB-22	.871	.008	113.70	<.001
Cognitive Avoidance Questionnaire	X13	Thought Suppression	RZHB-1	.768	.013	61.05	<.001
Cognitive Avoidance Questionnaire	X13	Thought Suppression	RZHB-2	.852	.008	100.94	<.001
Cognitive Avoidance Questionnaire	X13	Thought Suppression	RZHB-5	.867	.008	109.46	<.001

Scale	Node	Factor / subscale	Item	Std. loading	SE	z	p
Cognitive Avoidance Questionnaire	X13	Thought Suppression	RZHB-6	.860	.008	104.07	<.001
Cognitive Avoidance Questionnaire	X13	Thought Suppression	RZHB-14	.840	.009	89.04	<.001
ADEXI	X14	Working Memory Difficulties	ADEXI-1	.609	.018	34.30	<.001
ADEXI	X14	Working Memory Difficulties	ADEXI-2	.728	.013	56.00	<.001
ADEXI	X14	Working Memory Difficulties	ADEXI-5	.774	.011	68.66	<.001
ADEXI	X14	Working Memory Difficulties	ADEXI-7	.775	.012	67.13	<.001
ADEXI	X14	Working Memory Difficulties	ADEXI-8	.821	.010	84.91	<.001
ADEXI	X14	Working Memory Difficulties	ADEXI-9	.771	.011	67.25	<.001
ADEXI	X14	Working Memory Difficulties	ADEXI-11	.850	.008	106.81	<.001
ADEXI	X14	Working Memory Difficulties	ADEXI-12	.852	.008	111.28	<.001
ADEXI	X14	Working Memory Difficulties	ADEXI-13	.840	.009	97.56	<.001
ADEXI	X15	Inhibition Difficulties	ADEXI-3	.596	.019	31.43	<.001
ADEXI	X15	Inhibition Difficulties	ADEXI-4	.735	.014	52.24	<.001
ADEXI	X15	Inhibition Difficulties	ADEXI-6	.782	.013	60.01	<.001
ADEXI	X15	Inhibition Difficulties	ADEXI-10	.794	.013	61.32	<.001
ADEXI	X15	Inhibition Difficulties	ADEXI-14	.731	.014	50.71	<.001
Child Psychological Abuse Scale	X16	Intimidation	XLND-1	.634	.018	34.42	<.001
Child Psychological Abuse Scale	X16	Intimidation	XLND-2	.755	.013	56.35	<.001
Child Psychological Abuse Scale	X16	Intimidation	XLND-7	.835	.010	81.14	<.001
Child Psychological Abuse Scale	X16	Intimidation	XLND-9	.858	.010	90.18	<.001
Child Psychological Abuse Scale	X16	Intimidation	XLND-10	.834	.011	78.99	<.001
Child Psychological Abuse Scale	X16	Intimidation	XLND-14	.823	.011	75.12	<.001
Child Psychological Abuse Scale	X16	Intimidation	XLND-15	.824	.012	70.11	<.001
Child Psychological Abuse Scale	X17	Neglect	XLND-3	.712	.016	43.97	<.001
Child Psychological Abuse Scale	X17	Neglect	XLND-5	.868	.010	83.53	<.001
Child Psychological Abuse Scale	X17	Neglect	XLND-18	.816	.012	68.08	<.001
Child Psychological Abuse Scale	X17	Neglect	XLND-19	.761	.014	53.58	<.001
Child Psychological Abuse Scale	X17	Neglect	XLND-20	.832	.011	72.38	<.001
Child Psychological Abuse Scale	X17	Neglect	XLND-21	.809	.013	63.67	<.001
Child Psychological Abuse Scale	X18	Denigration	XLND-4	.822	.011	76.60	<.001
Child Psychological Abuse Scale	X18	Denigration	XLND-6	.814	.011	72.59	<.001
Child Psychological Abuse Scale	X18	Denigration	XLND-13	.845	.010	82.91	<.001

Scale	Node	Factor / subscale	Item	Std. loading	SE	z	p
Child Psychological Abuse Scale	X18	Denigration	XLND-16	.845	.011	79.97	<.001
Child Psychological Abuse Scale	X19	Interference	XLND-8	.783	.014	56.82	<.001
Child Psychological Abuse Scale	X19	Interference	XLND-11	.826	.012	68.93	<.001
Child Psychological Abuse Scale	X19	Interference	XLND-12	.838	.011	76.34	<.001
Child Psychological Abuse Scale	X19	Interference	XLND-17	.857	.011	75.30	<.001
Child Psychological Abuse Scale	X20	Indulgence	XLND-22	.927	.023	40.83	<.001
Child Psychological Abuse Scale	X20	Indulgence	XLND-23	.800	.023	34.78	<.001

Note. All reported loadings are standardized. p values below .001 are displayed as <.001.

Table S6. Gender Measurement Invariance Results for the Six Source Measures

Scale	Model	Converged	χ^2 (df)	CFI	TLI	RMSEA	SRMR	ΔCFI	ΔRMSEA	ΔSRMR	Scaled $\Delta\chi^2$ (Δdf)	LRT p
OGMQ	Configural	Yes	1300.90 (292)	.892	.873	.094	.048	—	—	—	—	—
OGMQ	Threshold	Yes	1335.89 (311)	.954	.949	.070	.048	.062	-.024	.000	24.13 (19)	.191
OGMQ	Metric	Yes	1315.47 (326)	.955	.953	.067	.049	.002	-.003	.000	14.49 (15)	.489
OGMQ	Scalar	Yes	1312.18 (341)	.956	.956	.065	.049	.001	-.002	.000	52.14 (15)	<.001
RRS	Configural	Yes	2391.73 (412)	.882	.868	.105	.045	—	—	—	—	—
RRS	Threshold	Yes	2421.75 (434)	.959	.957	.082	.045	.078	-.023	.000	17.88 (22)	.713
RRS	Metric	Yes	2401.96 (453)	.960	.959	.079	.045	.001	-.003	.000	45.20 (19)	<.001
RRS	Scalar	Yes	2407.35 (472)	.961	.961	.078	.045	.000	-.002	.000	94.25 (19)	<.001
AAQ-II	Configural	Yes	317.66 (28)	.963	.944	.128	.027	—	—	—	—	—
AAQ-II	Threshold	Yes	331.33 (56)	.989	.992	.085	.027	.026	-.043	.000	35.91 (28)	.145
AAQ-II	Metric	Yes	283.09 (62)	.991	.994	.072	.028	.002	-.013	.000	15.13 (6)	.019
AAQ-II	Scalar	Yes	268.85 (68)	.992	.995	.066	.028	.001	-.007	.000	19.70 (6)	.003
Cognitive Avoidance Questionnaire	Configural	Yes	3406.98 (530)	.900	.887	.100	.039	—	—	—	—	—
Cognitive Avoidance Questionnaire	Threshold	Yes	3448.02 (580)	.966	.965	.085	.039	.066	-.014	.000	47.56 (50)	.572
Cognitive Avoidance Questionnaire	Metric	Yes	3387.05 (600)	.967	.967	.083	.039	.001	-.003	.000	32.33 (20)	.040
Cognitive Avoidance Questionnaire	Scalar	Yes	3291.22 (620)	.968	.969	.080	.039	.001	-.003	.000	82.94 (20)	<.001
ADEXI	Configural	Yes	826.88 (152)	.945	.935	.081	.038	—	—	—	—	—
ADEXI	Threshold	Yes	859.31 (180)	.975	.975	.074	.038	.030	-.006	.000	37.18 (28)	.115
ADEXI	Metric	Yes	943.78 (192)	.973	.974	.076	.039	-.003	.001	.001	93.36 (12)	<.001
ADEXI	Scalar	Yes	959.59 (204)	.972	.975	.074	.039	-.000	-.002	-.000	56.42 (12)	<.001
Child Psychological Abuse Scale	Configural	No	—	—	—	—	—	—	—	—	—	—
Child Psychological Abuse Scale	Threshold	Yes	3954.67 (486)	.925	.922	.102	.067	—	—	—	—	—
Child Psychological Abuse Scale	Metric	Yes	3888.31 (504)	.927	.926	.099	.067	.002	-.003	.000	31.35 (18)	.026
Child Psychological Abuse Scale	Scalar	No	—	—	—	—	—	—	—	—	—	—

Note. Ordered-categorical multigroup models progressed from configural to threshold, metric, and scalar constraints. ΔCFI, ΔRMSEA, and ΔSRMR are changes from the immediately preceding model. Scaled $\Delta\chi^2$ tests compare each model with the preceding step. The complete sequence was not evaluable for the Child Psychological Abuse Scale because the configural and scalar models did not converge successfully. Approximate fit changes from threshold through metric and scalar constraints were interpreted together with scaled chi-square difference tests rather than as a mechanical pass/fail rule.

Table S7. OGM-Specific Conditional Edges and Connectivity of CaR-FA-X-Related Processes

Panel A. Primary NPN direct OGM edges and NPN/Spearman summary connectivity

Candidate process	NPN X1	NPN X2	NPN X3	NPN X4	NPN signed	NPN absolute	NPN nonzero	Spearman signed	Spearman absolute	Spearman nonzero
Working Memory Difficulties	.065	0.000	0.000	0.000	.065	.065	1	.079	.079	2
Symptom Rumination	.028	.027	.002	0.000	.058	.058	3	.052	.052	3
Experiential Avoidance	0.000	0.000	-.002	-.007	-.009	.009	2	-.005	.005	1

Note. X1 = Spatiotemporal Construction; X2 = Controlled Construction; X3 = Contextual Elaboration; X4 = Emotional Elaboration. Signed connectivity is the sum of the four regularized partial edge weights linking each candidate process to X1–X4; absolute connectivity is the sum of their absolute values. These indices are descriptive and do not represent mediation or causal effects.

Panel B. Spearman sensitivity direct OGM edges

Candidate process	X1	X2	X3	X4	Signed	Absolute	Nonzero
Working Memory Difficulties	.072	0.000	.006	0.000	.079	.079	2
Symptom Rumination	.036	.013	.003	0.000	.052	.052	3
Experiential Avoidance	0.000	0.000	0.000	-.005	-.005	.005	1

Table S8. Goldbricker-Identified Redundancy Candidate Pairs

Pair	Node 1	Variable 1	Node 2	Variable 2	Community	Prop. different	Pearson r	Spearman ρ	Decision
GB1	X3	Contextual Elaboration	X1	Spatiotemporal Construction	OGM	.111	.734	.694	Retained in primary network; included in PC1-reduced sensitivity component
GB4	X13	Thought Suppression	X12	Avoidance of Threatening Stimuli	Cognitive Avoidance	.167	.846	.847	Retained in primary network; included in PC1-reduced sensitivity component
GB2	X10	Transformation of Images into Thoughts	X9	Thought Substitution	Cognitive Avoidance	.167	.820	.828	Retained in primary network; included in PC1-reduced sensitivity component
GB3	X13	Thought Suppression	X10	Transformation of Images into Thoughts	Cognitive Avoidance	.167	.784	.801	Retained in primary network; included in PC1-reduced sensitivity component
GB5	X7	Reflective Pondering	X6	Brooding	Ruminative Responses	.222	.816	.809	Retained in primary network; included in PC1-reduced sensitivity component

Note. All five candidate pairs occurred within their predefined theoretical communities. All original nodes were retained in the primary 20-node network; connected within-community components were summarized by PC1 only in the separate redundancy-reduced sensitivity analysis.

Table S9. FDR-Adjusted Node-Centrality Differences Between Male and Female Networks in the Primary NPN Analysis

Node	Variable	Metric	Male	Female	Male − Female	Raw p	FDR q (40-test family)
X1	Spatiotemporal Construction	Strength	.905	.872	.034	.612	.818
X1	Spatiotemporal Construction	Expected Influence	.905	.768	.138	.031	.138
X2	Controlled Construction	Strength	.667	.716	-.050	.431	.713
X2	Controlled Construction	Expected Influence	.667	.676	-.009	.878	.991
X3	Contextual Elaboration	Strength	1.090	1.056	.035	.613	.818
X3	Contextual Elaboration	Expected Influence	1.090	1.007	.084	.224	.471
X4	Emotional Elaboration	Strength	.937	.936	.001	.991	.991
X4	Emotional Elaboration	Expected Influence	.887	.752	.135	.057	.228
X5	Symptom Rumination	Strength	1.255	1.340	-.085	.261	.502
X5	Symptom Rumination	Expected Influence	1.255	1.340	-.085	.264	.502
X6	Brooding	Strength	.906	.871	.035	.552	.789
X6	Brooding	Expected Influence	.906	.835	.071	.195	.471
X7	Reflective Pondering	Strength	.901	.862	.039	.503	.746
X7	Reflective Pondering	Expected Influence	.901	.814	.087	.123	.446
X8	Experiential Avoidance	Strength	.715	.865	-.150	.145	.446
X8	Experiential Avoidance	Expected Influence	.630	.865	-.235	.005	.050
X9	Thought Substitution	Strength	.990	.905	.085	.199	.471
X9	Thought Substitution	Expected Influence	.990	.905	.085	.197	.471
X10	Transformation of Images into Thoughts	Strength	1.059	.885	.174	.008	.053
X10	Transformation of Images into Thoughts	Expected Influence	1.059	.885	.174	.008	.053
X11	Distraction	Strength	.985	1.080	-.096	.142	.446
X11	Distraction	Expected Influence	.985	1.034	-.049	.446	.713
X12	Avoidance of Threatening Stimuli	Strength	1.103	1.102	.002	.985	.991
X12	Avoidance of Threatening Stimuli	Expected Influence	1.103	1.083	.020	.754	.887
X13	Thought Suppression	Strength	.941	.997	-.056	.404	.702
X13	Thought Suppression	Expected Influence	.941	.997	-.056	.377	.685
X14	Working Memory Difficulties	Strength	.892	.923	-.030	.677	.821
X14	Working Memory Difficulties	Expected Influence	.892	.866	.027	.674	.821
X15	Inhibition Difficulties	Strength	.777	.599	.177	.018	.090
X15	Inhibition Difficulties	Expected Influence	.733	.550	.183	.010	.057
X16	Intimidation	Strength	1.116	1.118	-.001	.985	.991
X16	Intimidation	Expected Influence	1.116	1.118	-.001	.987	.991
X17	Neglect	Strength	1.047	.970	.077	.222	.471
X17	Neglect	Expected Influence	1.047	.970	.077	.222	.471
X18	Denigration	Strength	1.024	1.015	.009	.925	.991
X18	Denigration	Expected Influence	.942	.985	-.043	.485	.745
X19	Interference	Strength	.942	.698	.244	.003	.040
X19	Interference	Expected Influence	.932	.641	.291	<.001	.020
X20	Indulgence	Strength	.371	.340	.031	.645	.821

Node	Variable	Metric	Male	Female	Male − Female	Raw p	FDR q (40-test family)
X20	Indulgence	Expected Influence	.371	.212	.159	<.001	.020

Note. Male n = 822; female n = 542. Raw permutation p values were reconstructed from the NCT permutation distributions. FDR correction was applied across the 40 requested node-by-centrality tests (20 nodes × Strength and Expected Influence). These comparisons were secondary and exploratory.

Figure S1. Spearman Sensitivity Analyses

Panel A. 20-node Spearman EBICglasso sensitivity network.

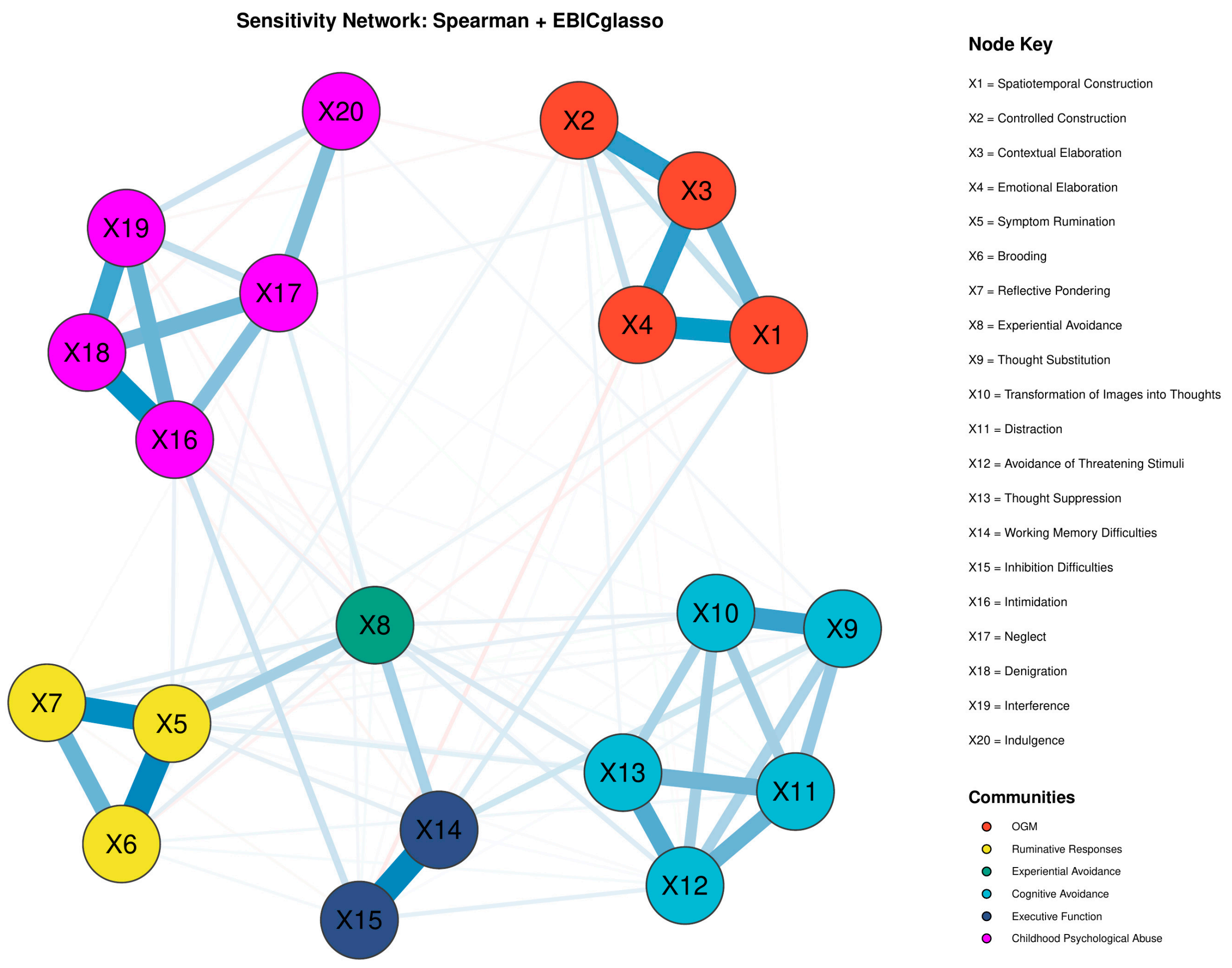

Figure S1. Spearman Sensitivity Analyses (continued)

Panel B. Comparison of edge weights from the primary NPN and Spearman sensitivity networks.

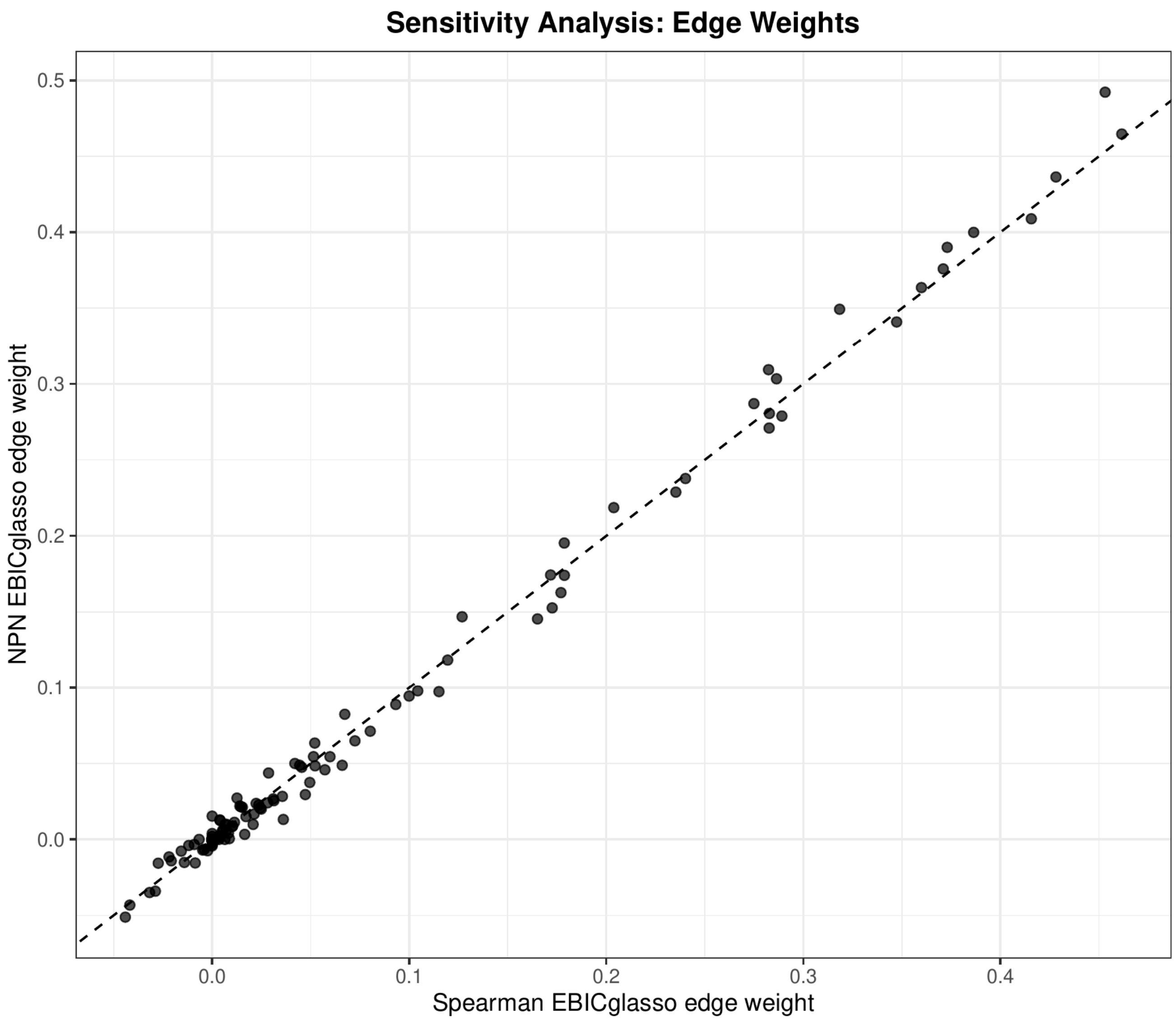

Figure S2. Nonparametric Bootstrap 95% Confidence Intervals for Edge Weights

Larger edge estimates were generally more precisely estimated than weak or near-zero edges.

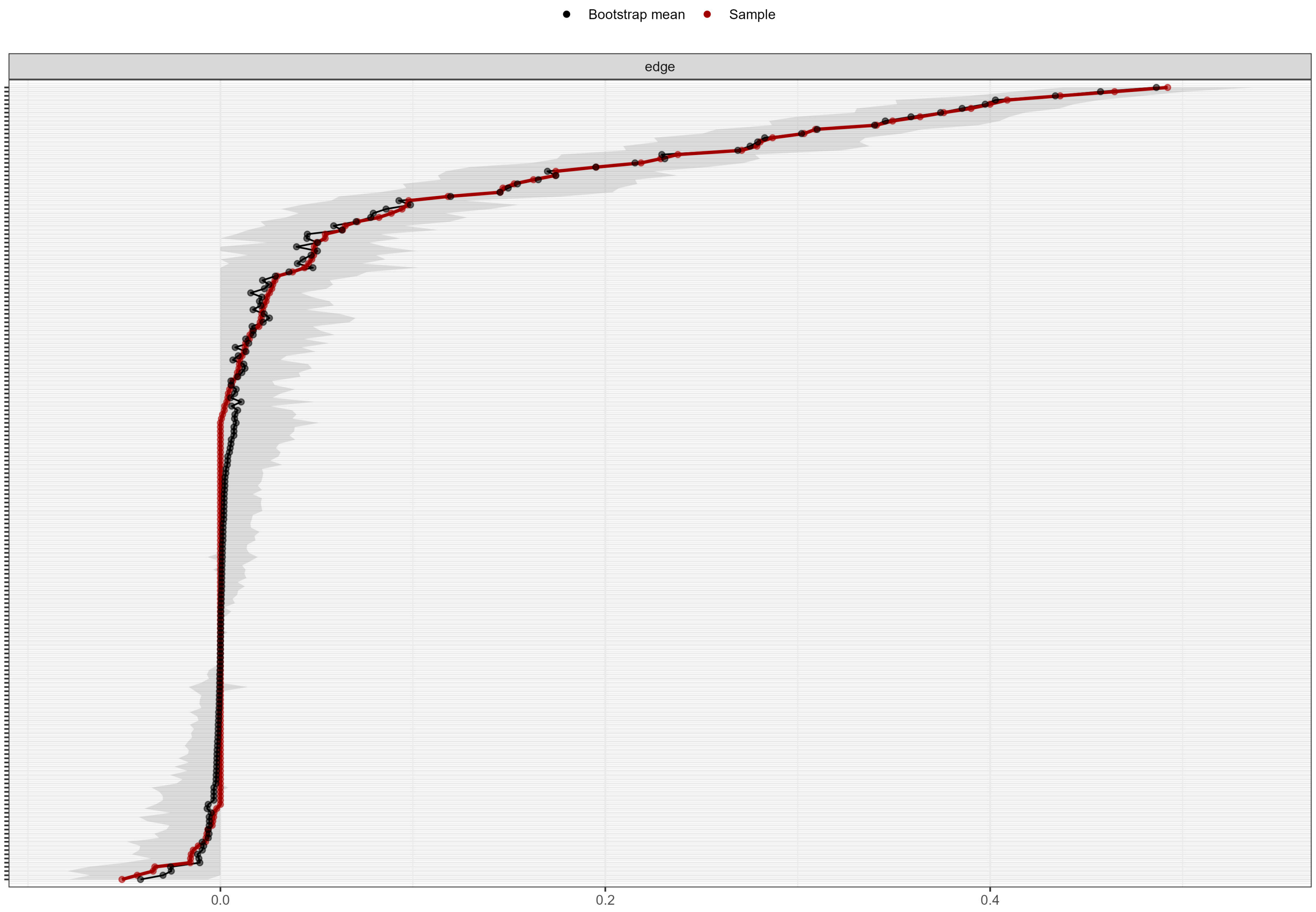

Figure S3. Bootstrap Difference Tests

Panel A. Pairwise difference test for edge weights.

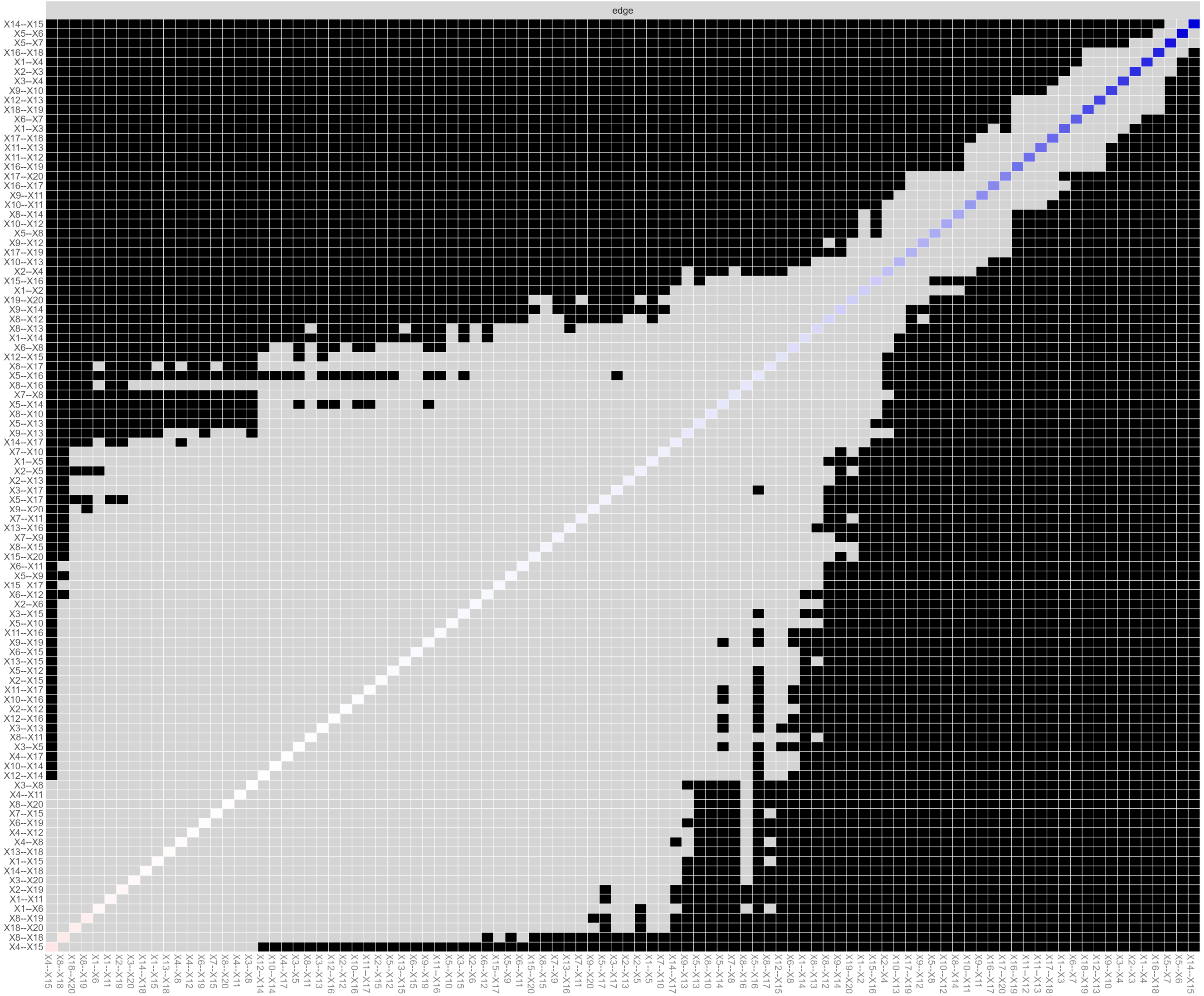

Edge labels use the original X1-X20 node codes; see the final node key for full English definitions.

Figure S3. Bootstrap Difference Tests (continued)

Panel B. Pairwise difference test for Expected Influence.

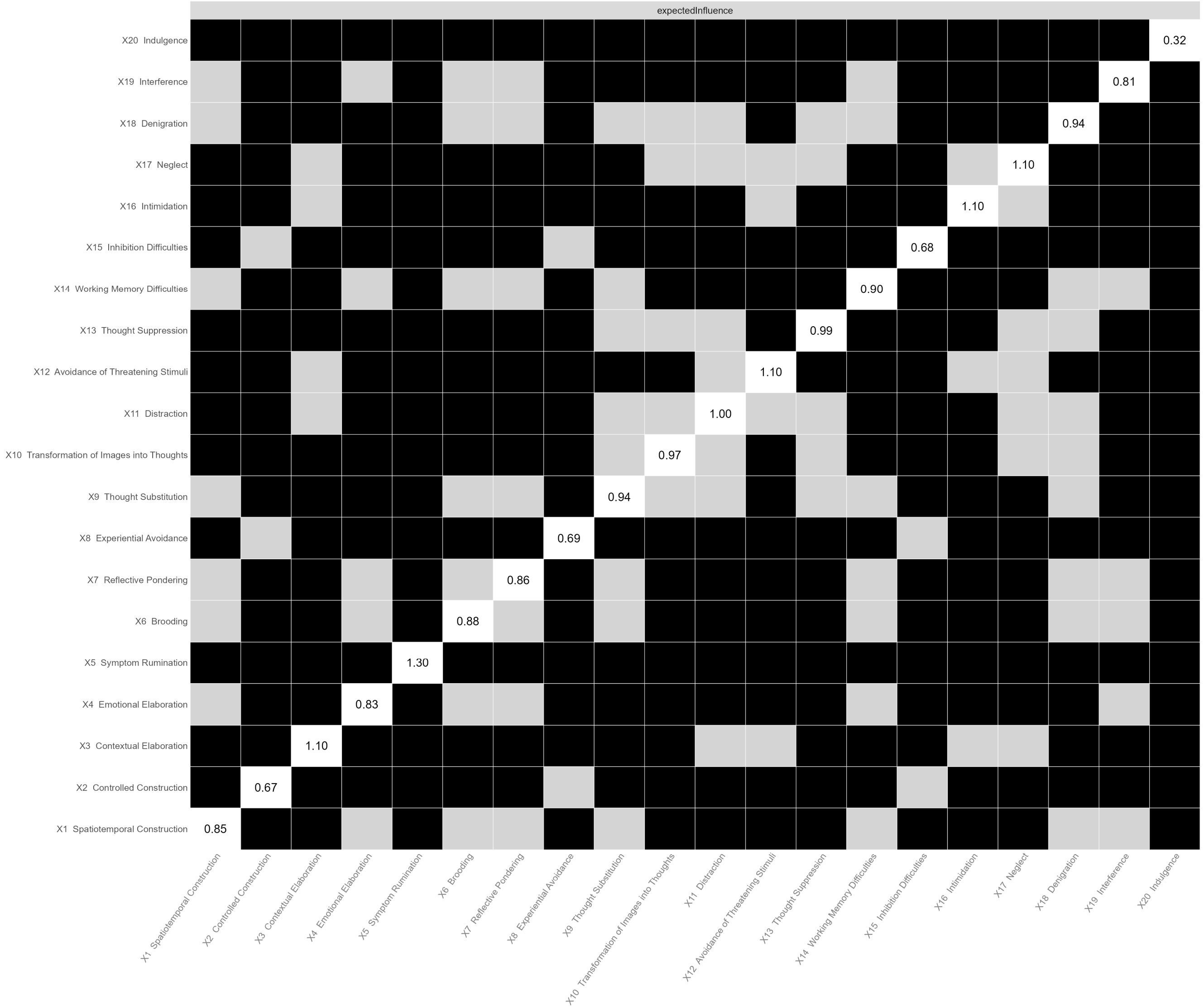

Figure S3. Bootstrap Difference Tests (continued)

Panel C. Pairwise difference test for Bridge Expected Influence.

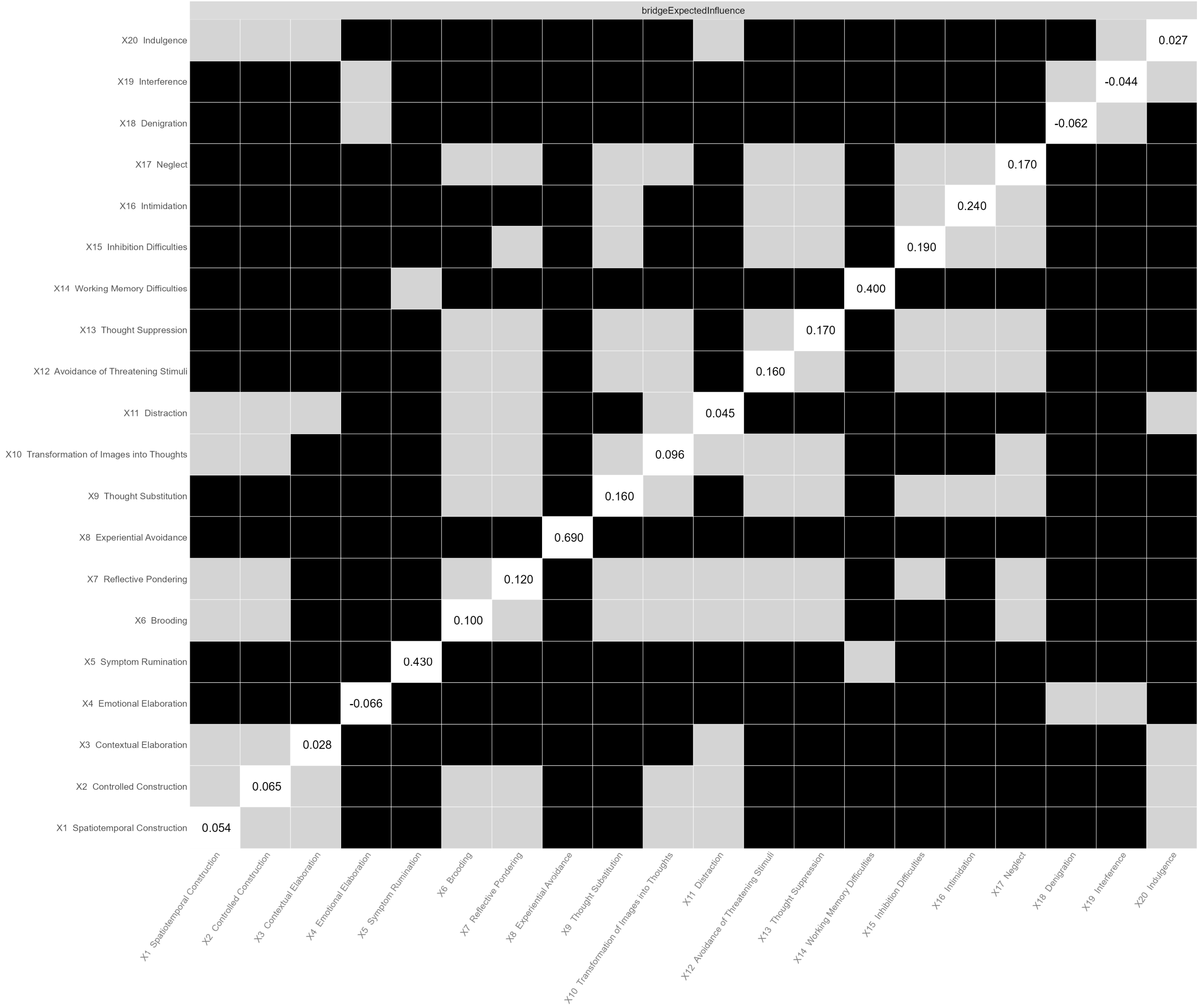

Figure S4. Goldbricker-Reduced Sensitivity Analyses

Panel A. Redundancy-reduced NPN network with predictability rings.

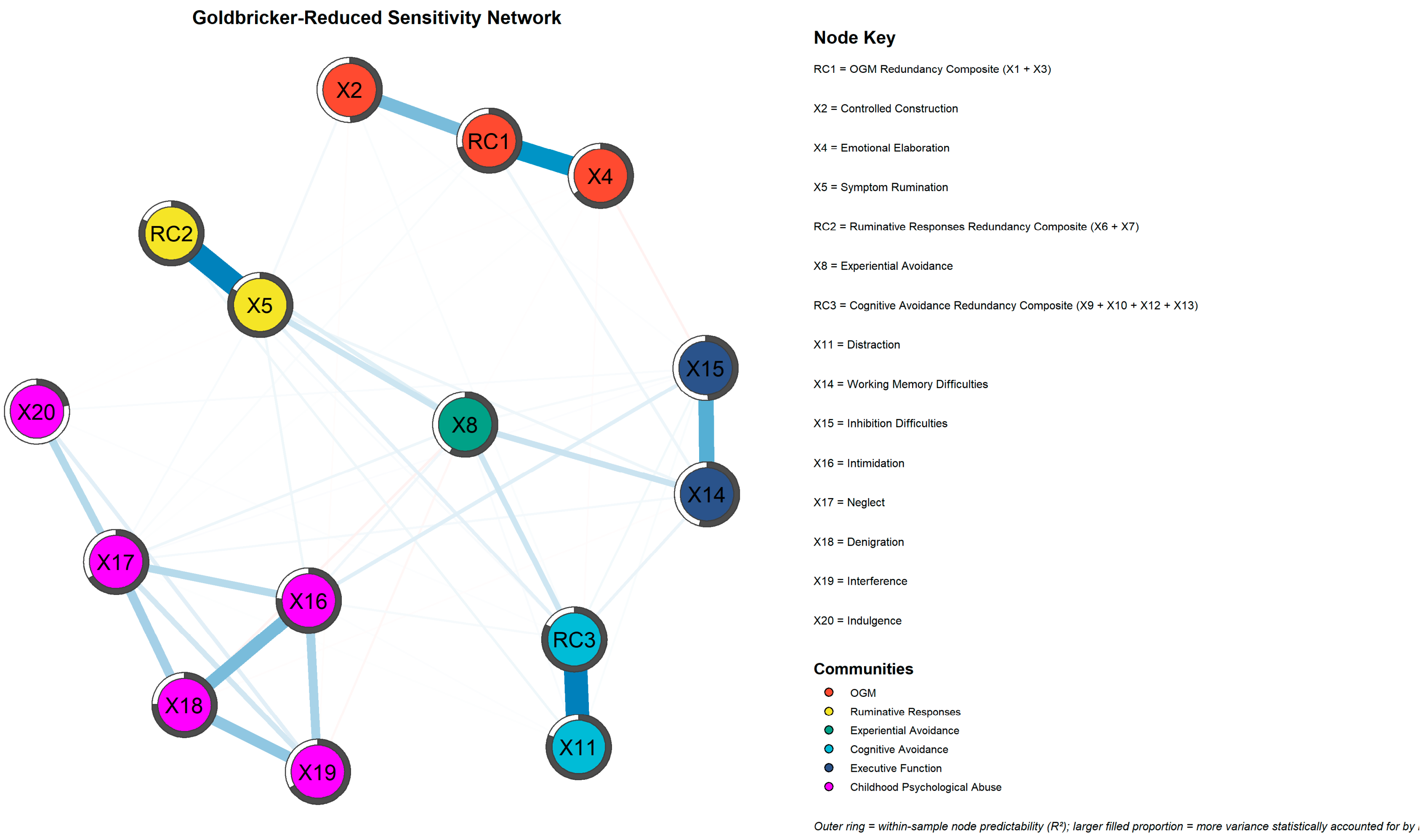

Figure S4. Goldbricker-Reduced Sensitivity Analyses (continued)

Panel B. Strength and Expected Influence in the reduced network.

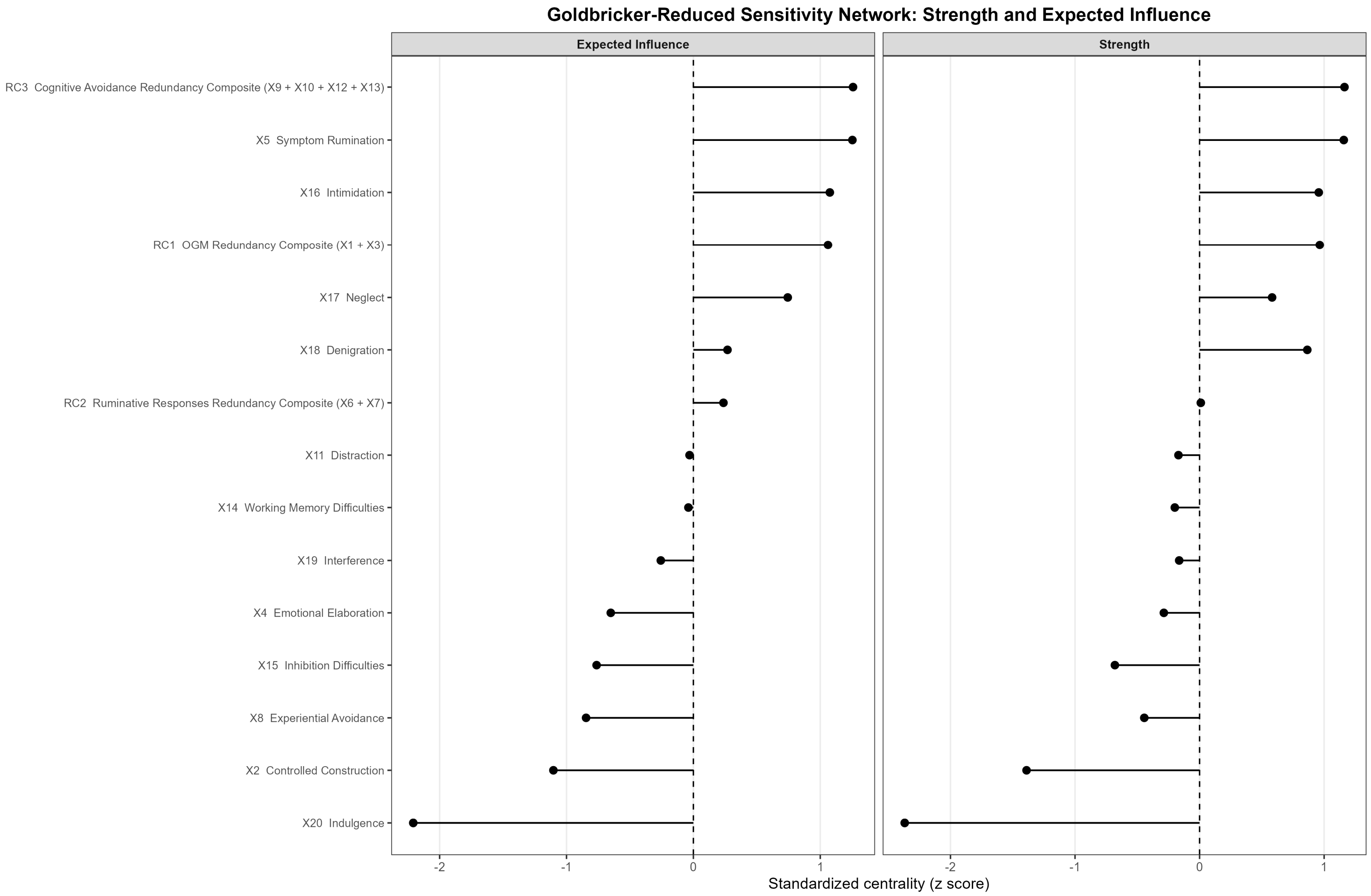

Figure S4. Goldbricker-Reduced Sensitivity Analyses (continued)

Panel C. Primary 20-node versus reduced-network common-node edge weights.

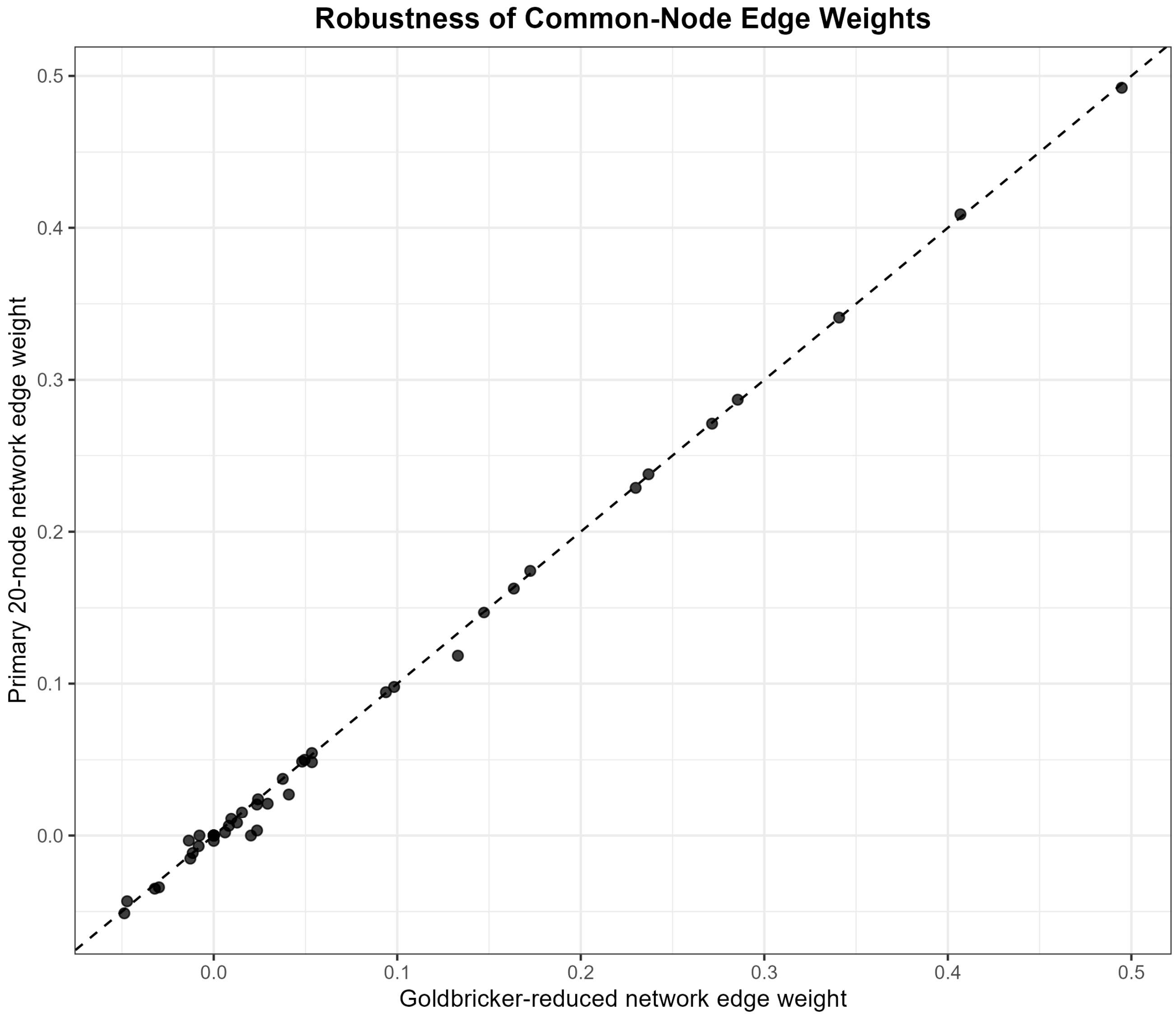

Figure S4. Goldbricker-Reduced Sensitivity Analyses (continued)

Panel D. Primary versus reduced-network common-node Strength, Expected Influence, and Bridge Expected Influence.

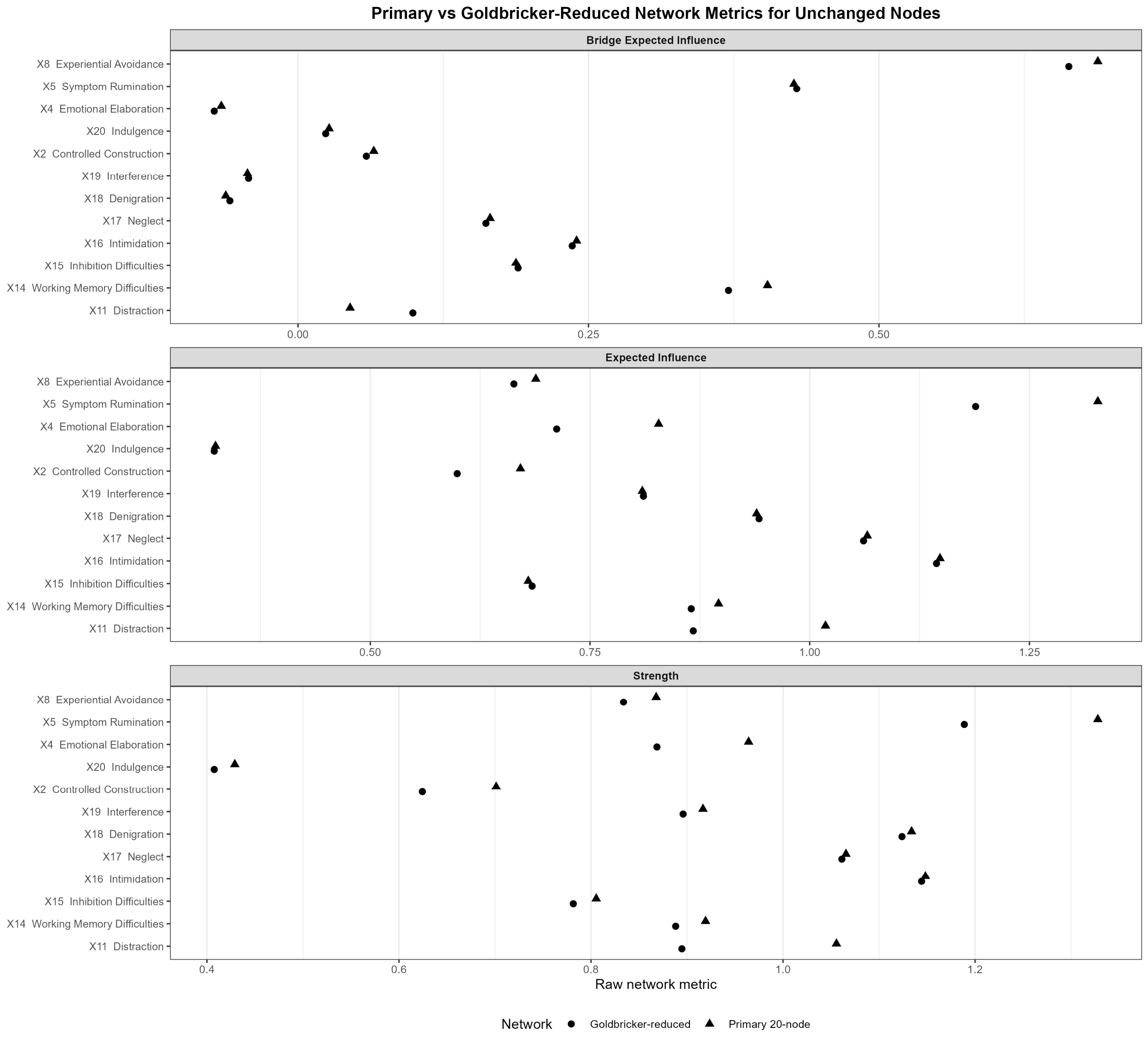

Figure S5. Gender-Specific Primary NPN Networks

Panel A. Male network (n = 822).

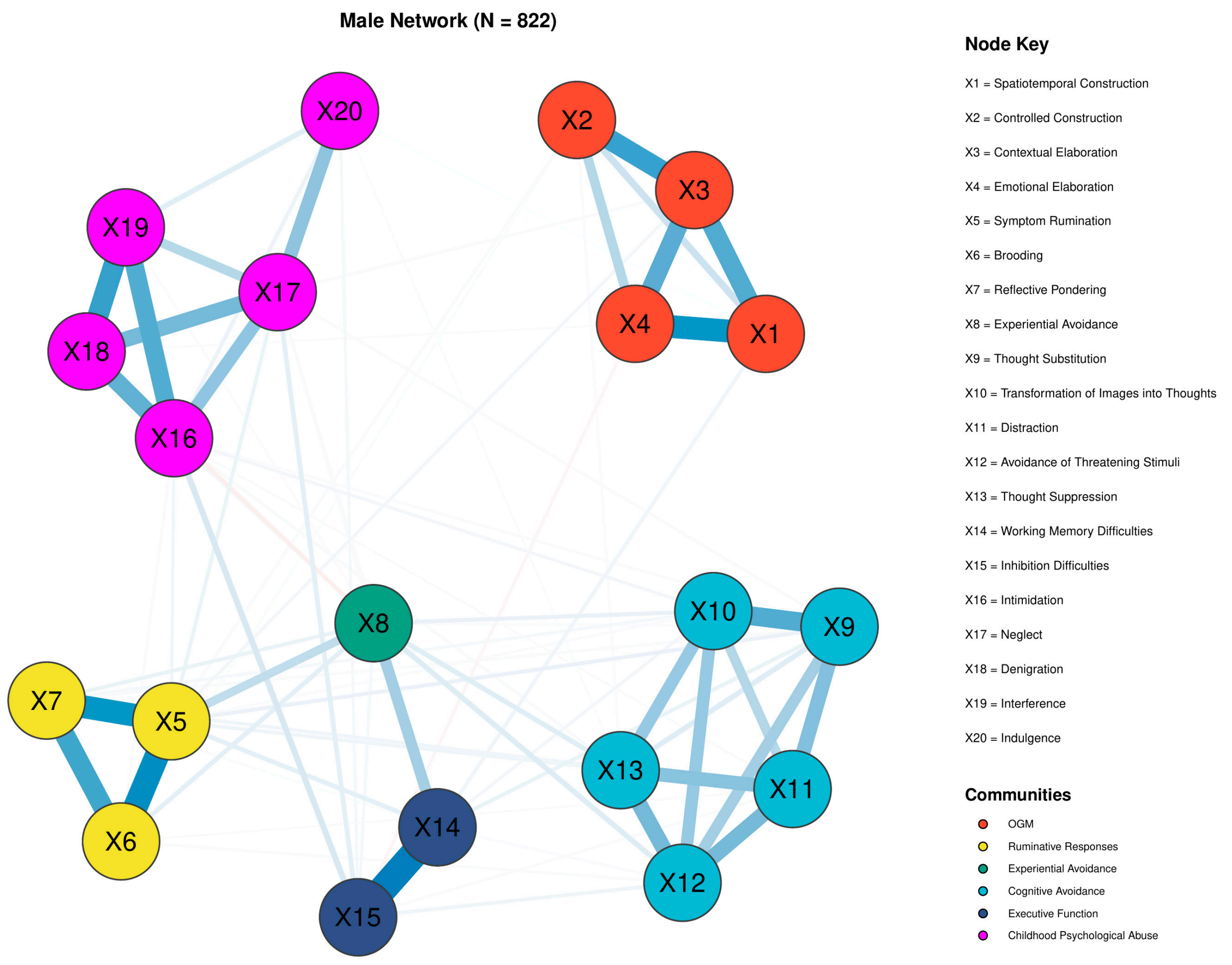

Figure S5. Gender-Specific Primary NPN Networks (continued)

Panel B. Female network (n = 542).

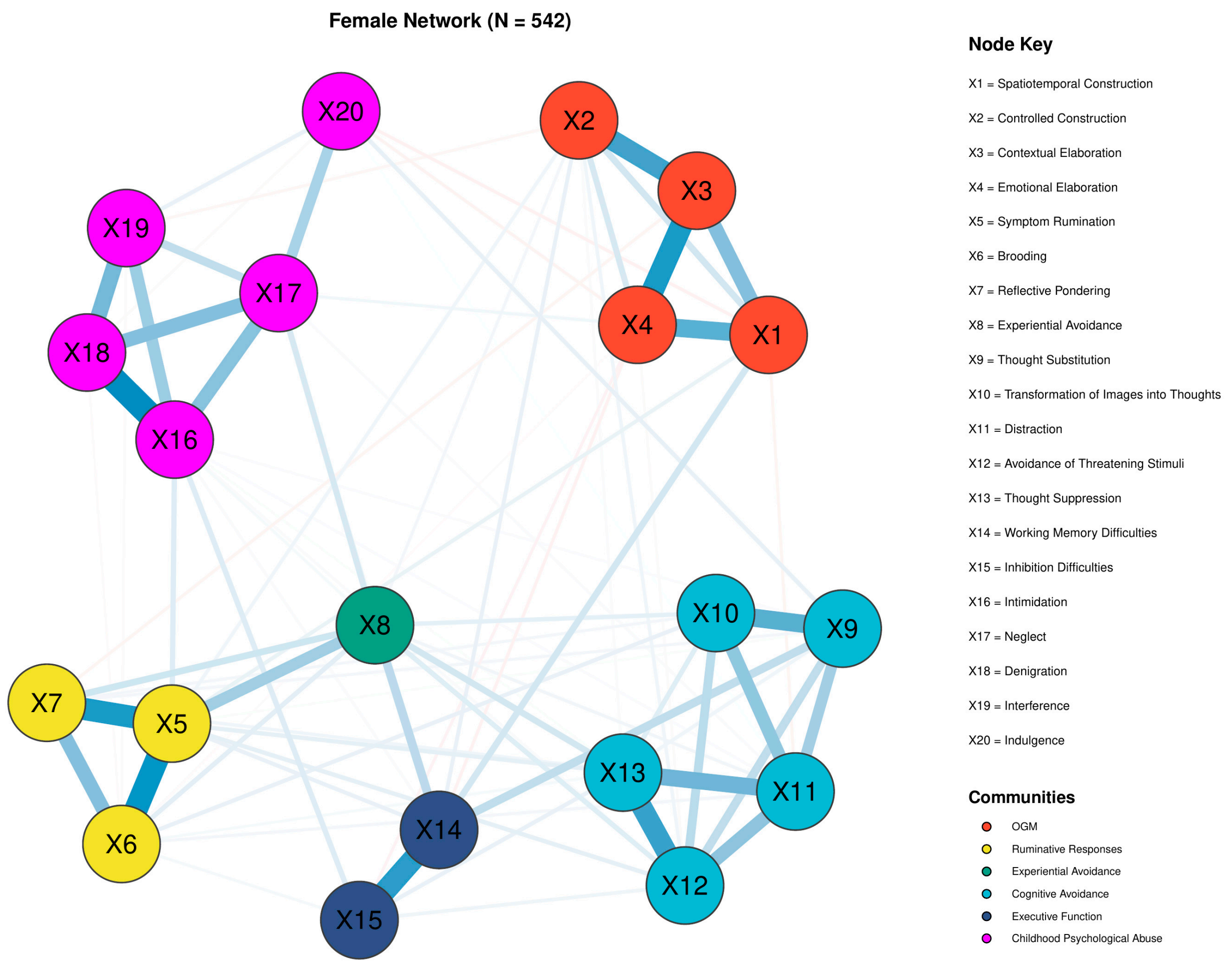

Figure S6. OGM-Specific Conditional Connectivity in the Primary NPN Network

Regularized partial edges linking Working Memory Difficulties, Symptom Rumination, and Experiential Avoidance to the four OGM nodes.

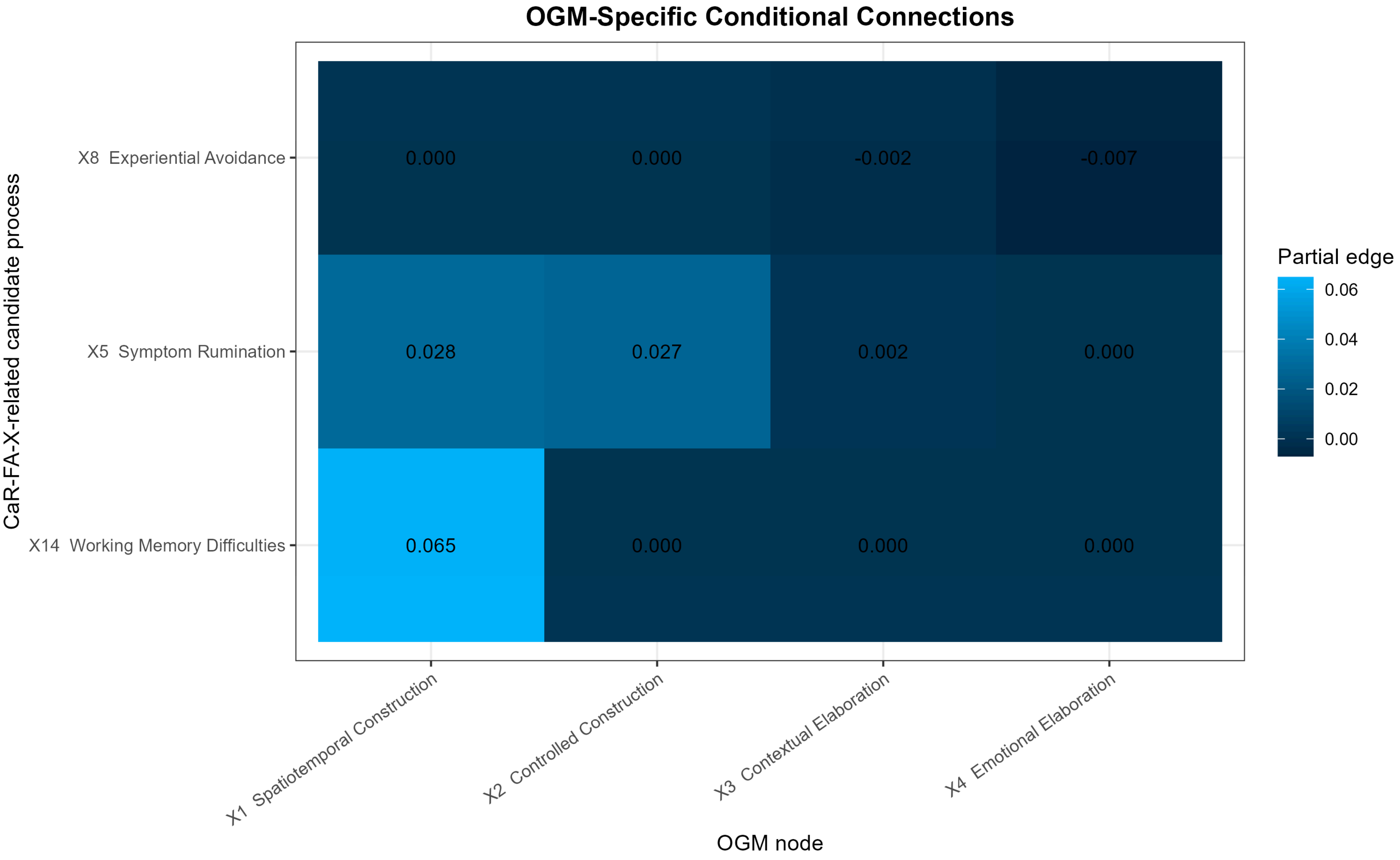

Supplementary Data

Supplementary Data S1. Full FDR-adjusted edge-specific Network Comparison Test results are provided as two machine-readable CSV files: primary NPN (S1A) and Spearman sensitivity (S1B), each containing all 190 edge comparisons.
